# Integrating Functional and Phylogenetic Diversity to Assess Bird Community Assembly Along the Major Rivers of Hainan Island, South China

**DOI:** 10.1002/ece3.70962

**Published:** 2025-02-10

**Authors:** Sidan Lin, Wei Liang

**Affiliations:** ^1^ Ministry of Education Key Laboratory for Ecology of Tropical Islands, Key Laboratory of Tropical Animal and Plant Ecology of Hainan Province, College of Life Sciences Hainan Normal University Haikou China

**Keywords:** community assembly, environmental filtering, functional diversity, phylogenetic diversity, river bird community

## Abstract

Understanding the mechanisms associated with community assembly can contribute to explaining the formation and maintenance of biodiversity patterns. In this study, we used the line transect method to survey breeding birds along the three major rivers of the Nandu River, Changhua River and Wanquan River on Hainan Island, south China. The patterns of community assembly were subsequently assessed by integrating functional and phylogenetic diversity, whereas environmental factors and interspecific competition intensity were incorporated to determine whether community assembly in these rivers is driven by environmental filtering or interspecific competition. Our findings revealed that bird communities within rivers were characterized by an overall slight clustering (i.e., more similar species), with the upper reaches of the Changhua River and the lower reaches of the Nandu River showing over‐dispersion, whereas the lower, middle, and upper reaches of the Wanquan River all showed clustering. Altitude and the human influence index were identified as the main factors driving bird community assembly within the three major rivers. Notably, for bird communities along different river reaches, the integration of functional and phylogenetic diversity prevented the mis‐classification of over‐dispersion or clustering in community structure caused by traits with weak phylogenetic signals, or the observation of traits unrelated to community assembly patterns. This empirical study demonstrates the importance of integrating functional and phylogenetic diversity, which not only contributes to gaining an understanding of the mechanisms underlying the development of community assemblies but also facilitates a determination of the extents to which function and phylogeny contribute to shaping the patterns of communities.

## Graphical abstract

Bird communities within rivers were characterized by an overall slight clustering. Altitude and the human influence index were identified as the main factors driving bird community assembly within the three major rivers of Hainan Island. This study demonstrates the importance of integrating functional and phylogenetic diversity.

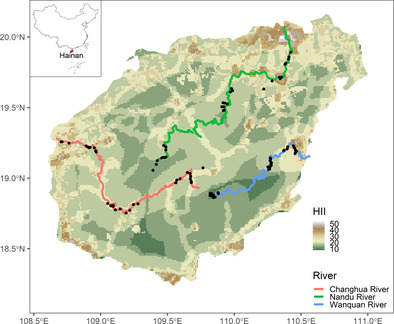

## 1 Introduction

The diverse array of anthropogenic disturbances has led to alterations in riverine ecosystems worldwide (Reid et al. 2018). Empirical analyses reveal that the escalating pressure from human interference has resulted in the loss of biodiversity among various biological taxa and trophic levels within river ecosystems (Moi et al. 2022). Riverine ecosystems possess not only ecological, landscape, and social value but also encompass a relatively intact habitat structure that includes rivers, beaches, gravel beds, and grasslands, providing crucial breeding habitats for numerous species (Mitsch and Gosselink 2000). For instance, river wetlands offer birds essential foraging and resting sites (Wilson 2010), whereas urban developments and bridges encroaching upon rivers have led to the degradation of natural habitats for species such as birds (Rehman et al. 2021). Thus, the conservation of riverine ecosystems is vital for the protection of avian diversity. However, previous research has primarily focused on migratory birds in estuarine and coastal regions (e.g., China Coastal Waterbird Census Group et al. 2015; Kuwae et al. 2021; Lin and Ma 2024), with a notable absence of studies on bird diversity in inland freshwater river areas.

Niche theory (Chase and Leibold 2003) and neutral theory (Bell 2000; Hubbell 2001; Adler, HillerisLambers, and Levine 2007) provide a framework for understanding the formation and maintenance of bird community structures within river ecosystems. If the environmental conditions of river ecosystems (e.g., elevation, anthropogenic disturbance) selectively filter the regional species pool (habitat filtering), it is anticipated that the ecological niches of species within the community will converge (Diamond 1975; Keddy 1992; MacArthur 1958; Lavorel and Garnier 2002). Conversely, if interspecific interactions (e.g., competition) within the river ecosystem prevent similar species from coexisting in resource‐limited environments (limiting similarity), then niche differentiation is expected among community species (Gause 1934; MacArthur and Levins 1967; Wilson and Gitay 1995). Additionally, if the assembly of bird communities is a stochastic zero‐sum process influenced by species dispersal, reproduction, mortality, immigration, emigration, and extinction, then community similarity is likely to be primarily dictated by spatial distance (dispersal limitation) (Hubbell 2001; Condit et al. 2002). In contrast to niche theory, which emphasizes the impact of interspecific niche differences on community structure, neutral theory does not take interspecific niche differentiation as its starting point for studying community structure. Instead, neutral theory posits that all individuals within the same trophic level are ecologically equivalent, possessing identical statistical attributes, including probabilities of birth, death, immigration, and emigration, as well as speciation probabilities. According to neutral theory, community structure arises from stochastic processes such as random birth and death events, migration, and speciation, rather than from deterministic interactions driven by niche differentiation (Hubbell 2001). Thus, while niche theory underscores the importance of interspecific interactions and ecological specialization, neutral theory provides an alternative framework where species are functionally equivalent, and community assembly is primarily influenced by random ecological and demographic processes (Chave 2004). Assessing the diversity of bird communities and the impact of related factors can yield insights into the mechanisms behind the construction of riverine avian communities.

Historically, assessments of avian community similarity have been based on the presence‐absence and abundance of species (Diamond 1975; Gotelli 2000; Mason et al. 2005; Götzenberger et al. 2012). However, as phylogenetic and functional structure data have become increasingly comprehensive, researchers have begun to examine community assembly mechanisms through phylogenetic diversity and functional diversity (Cadotte et al. 2013; Song et al. 2022). Phylogenetic diversity (PD) reflects the evolutionary history and relationships among species (Faith 1992), while functional diversity (FD) indicates the occupation of ecological niches or the distribution patterns of species within niche space (Diaz et al. 1998). By comparing the phylogenetic or trait distribution patterns of actual communities with those of random communities, one can assess whether community phylogenetic or functional structures are clustered (more similar species) or divergent (more dissimilar species) (Keddy 1992; Webb et al. 2002; Kraft et al. 2007). However, relying solely on PD or FD may not accurately reflect community assembly mechanisms, as PD assumes traits are phylogenetically conserved (Du et al. 2017), while FD‐based approaches struggle to determine which traits (Weiss and Ray 2019) and how many traits (Mouillot et al. 2021) are relevant to community assembly. Consequently, recent studies have sought to integrate PD and FD into a unified framework, combining interspecific distances of phylogenetic and functional traits to establish functional‐phylogenetic distances (FPDist) (Cadotte et al. 2013). Compared to using PDist or FDist alone, FPDist offers a more robust estimate of overall functional differences among species, aiding in the elucidation of driving forces behind ecological processes (Cadotte et al. 2013). For example, Si et al. (2017) employed this method to investigate the effects of habitat loss and fragmentation on bird community assembly in Qiandao Lake, Zhejiang, China, validating the nonrandom processes outlined in the Theory of Island Biogeography. Thorn et al. (2016) applied this approach to examine changes in community assembly patterns across different biological taxa before and after the removal of downed wood, revealing that patterns varied according to reliance on downed wood for survival, with different weights assigned to FDist and PDist for various taxa. Nonetheless, no studies have yet integrated PD and FD to explore the mechanisms of bird community assembly in riverine environments.

In the present study, we used an integrated FD‐PD approach to investigate the assembly of bird communities associated with different rivers on Hainan Island, south China. As different rivers, we examined the upper, middle, and lower reaches of the Nandu River, Changhua River and Wanquan River, along which, to the best of our knowledge, there have been no comprehensive background surveys of bird diversity conducted to date. Our primary aims in this study were (1) to characterize the bird community assemblies associated with different rivers, (2) determine the factors driving bird community assembly in different rivers, (3) to identify the extent to which FDist and PDist contribute to community assembly, and (4) establish whether approaches based on integrated FDist‐PDist are superior to FDist or PDist considered separately.

## 2 Methods

### 2.1 Study Area

**FIGURE 1:**
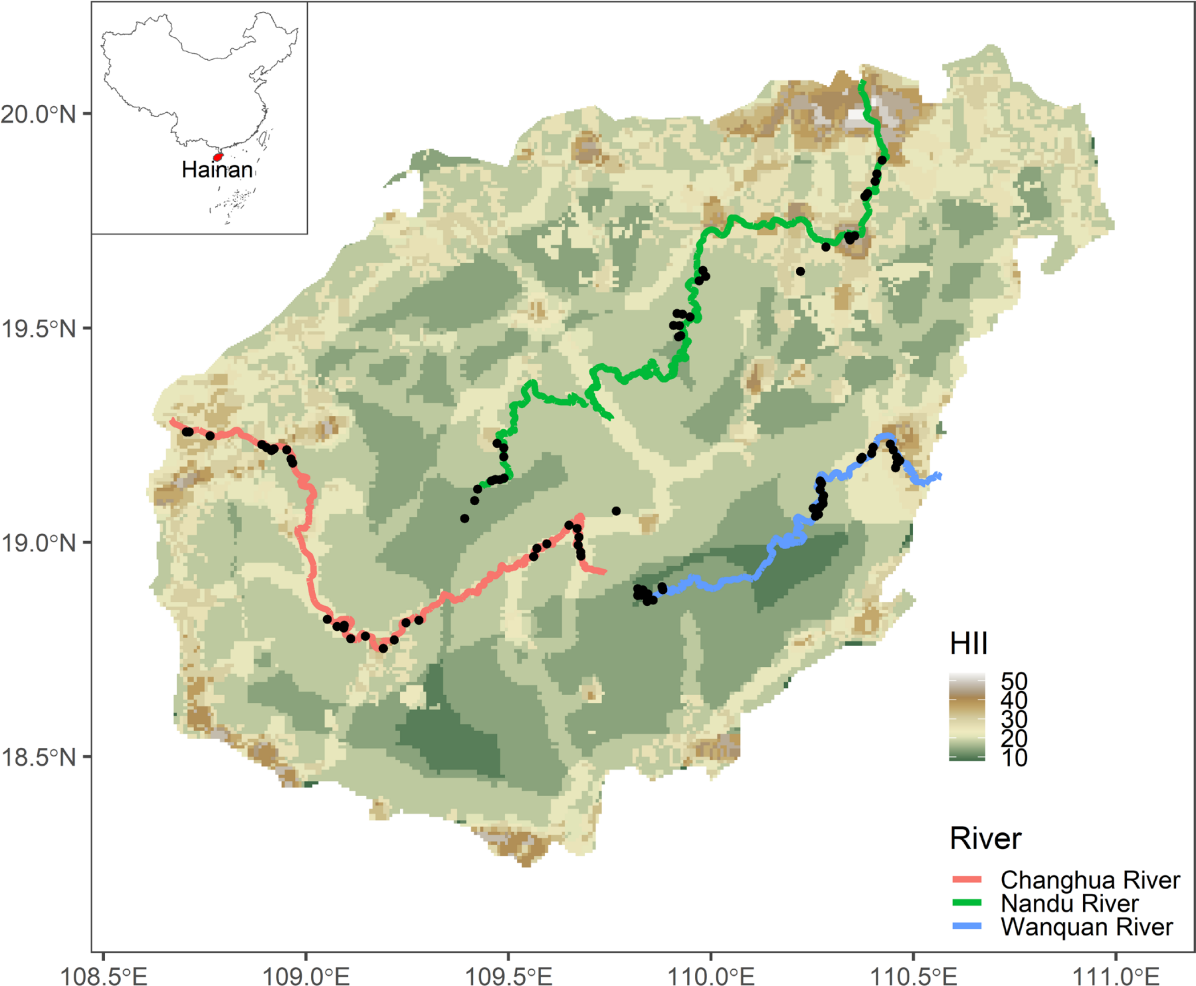
Location of the study area, rivers, and bird survey sites. The base map colors represent the Human Influence Index (HII).

Hainan Island, located in the south of China (18°10′–20°10′ N, 108°37′–111°03′ E), is the second largest island in China, with a tropical oceanic climate which is warm and hot throughout the year, with an average annual temperature of between 23°C and 26°C (Li et al. 2013). The entire island receives abundant rainfall, with an annual average of > 1600 mm, larger amounts of which fall in the east than in the west. Accordingly, the central and eastern parts of the island tend to be relatively wet, whereas the southwestern coastal areas are relatively dry. The distribution of rainfall is uneven across the different seasons, with less rain in winter and spring (dry season), and larger amounts in the summer and autumn (rain season) (Rao et al. 2017; Liu and Liang 2021). The terrain of the peripheral areas of Hainan Island is generally low and flat, whereas that of the center, in which Wuzhi and Yinggeling Mountains form the core of the uplift is contrastingly elevated. The islands rivers flow into the sea from the central mountainous or hilly areas through the surrounding lowland areas according to the terrain, forming a radial island river system (Rao et al. 2017). The three main watersheds on Hainan Island, all originating in the central mountainous areas, are those of the Nandu River (333.8 km, flowing northward), Changhua River (232 km, flowing westward) and Wanquan River (157 km, flowing southward). We employed digital elevation maps to discern ridges and valleys, delineate river channels and watershed divides, and subsequently divided the river into approximate thirds: upper, middle, and lower reaches (Figure 1).

### 2.2 Bird Community Data

To investigate the structural composition of bird communities along the Nandu River, Changhua River and Wanquan River, we conducted field surveys of birds during the breeding season in 2022 using a standard line transect method (Bibby et al. 1992). The three rivers were divided into lower, middle, and upper reaches (Figure 1). Owing to logistical constraints, we were unable to survey birds along the entire river. However, we established 10 transects in the upper, middle, and lower reaches of each river, giving a total of 90 transects and striving to ensure that our findings accurately reflect the composition of the local bird communities. Each transect was 1 km length and established close to the respective river banks (mean distance 1.4 km). Moreover, inter‐transects distances were greater than 1 km to ensure independence between transect counts. During surveys, we traveled along the transect at walking speed whilst observing and identify birds using binoculars (Swarovski NL 8.5 × 42). We documented the birds observed or heard within 100 m on either side of the transect line. The species and numbers of birds seen or heard on both sides of the transect were recorded, and the coordinates and elevation of the starting point of the transect were recorded using the mobile APP “2bulu.” Each survey was conducted between sunrise and 11:00 am at local Beijing time. No surveys were conducted during the middle of the day, as this corresponds with a period of low bird activity. Surveys were conducted only in good weather, avoiding rainy or windy conditions, which are also associated with low bird activity, and would thus influence detection rates. Each transect is surveyed only once. Abundance data collected for birds may be subject to false absences owing to inadequate survey design, and thus to minimize this bias, we aggregated bird species from the transects based on different rivers and reaches, and retained the community matrix of species presence or absence for subsequent analyses (i.e., a species was recorded as present if it was observed during surveys) (Table S1).

### 2.3 Environmental Data

Given that abiotic factors (habitat filtering) may contribute to niche similarity among species within a community, and that this is one of the mechanisms associated with community assembly (Kraft et al. 2015), we collected data pertaining to environmental variables to determine whether habitat filtering drives community assembly. As bird distribution may be influenced by climate and habitat (Renwick et al. 2012; Luck et al. 2013), we downloaded the following data: the 1981–2010 climate datasets for the global mean annual air temperature (TEMP) and annual precipitation amount (PREC) from the CHELSA database (Climatologies at high resolution for the earth's land/surface areas; http://chelsa‐climate.org/) (Karger et al. 2017); the May 2022 datasets for global normalized difference vegetation index (NDVI) and net primary productivity (NPP) from NEP (NASA Earth Observatory, https://earthobservatory.nasa.gov/global‐maps); and the 1995–2004 geographical datasets for the global human influence index (HII) from SEDAC (Socioeconomic Data And Application Center, https://sedac.ciesin.columbia.edu/data/set/wildareas‐v2‐human‐influence‐index‐geographic (Sanderson et al. 2002). The approximate location and altitude of the transects were expressed by averaging the coordinates and altitude of the transect start and end points. ArcGIS 10.2 (ESRI, Redlands, CA, USA) was used to extract the TEMP, PREC, NDVI, NPP, and HII values from the aforementioned dataset to the transects according to latitude and longitude. Finally, the transects were divided into nine groups according to river and reach, and then the TEMP, PREC, NDVI, NPP, HII, and altitude (ALT) values was averaged across transects in each group to represent the environmental variables for each reach (Table S2).

### 2.4 Community Phylogeny and Functional Traits

To obtain phylogenetic trees for our target birds, a total of 5000 phylogenetic trees were downloaded from BirdTree (http://birdtree.org/) to include the 105 species recorded in this survey, under the option of “Hackett All Species: a set of 10,000 trees with 9993 OTUs each” (Jetz et al. 2012). Using the TreeAnnonator software in the BEAST package (v2.6.7), we constructed a maximum clade credibility tree using mean node heights. The tree thus generated was used for subsequent phylogenetic analysis.

As functional traits, we selected the morphological traits, body mass, habitat, trophic level, lifestyle, and reproductive traits of the birds (Wilman et al. 2014; Tobias et al. 2022; Table S3), which reflect the resource use and niche differentiation of birds. For example, the beak is used by birds to capture and process food, and is associated with dietary niche and resource competition; the wings, tails, and legs are associated with locomotion, which reflects how birds move and forage within a habitat (Pigot et al. 2020). Body mass is also considered to be directly correlated with the resource use and trophic levels of animals, as well as the risk of extinction (Ding et al. 2013). Functional traits can give rise to a quantitative framework, which can contribute to gaining an understanding and predicting the mechanisms underlying species coexistence and ecological community assembly (Trisos et al. 2014; Pigot et al. 2018; Schleuning et al. 2020). For example, functional traits can be used to explain how trophic interaction networks regulate ecological processes, such as pest control, pollination, and seed dispersal (Bregman et al. 2015, 2016; Schleuning et al. 2015; Bender et al. 2019), as well as how ecological services corresponding to different functional traits (e.g., pollination and seed dispersal) are lost with a loss of biodiversity (Tobias et al. 2020; Weeks et al. 2022).

### 2.5 Diversity Measures

To assess the different dimensions of bird diversity (i.e., PD and FD), we integrated species functional traits and phylogenies using a method proposed by Cadotte et al. (2013). Initially, we calculated the functional distance (FDis) between species based on the functional traits (Gower 1966) using the function *gowdis* in the R package *FD* (Laliberté and Legendre 2010; Laliberté et al. 2014). The advantage of calculating the Gower distance between species lies in its ability to handle nominal traits (Laliberté and Legendre 2010). Principal coordinates analysis (PCoA) was performed on FDist data using the function *cmdscale* in the R package *vegan* (Oksanen et al. 2022). The first few principal axes with higher interpretability were obtained and adopted as the new FDist. Phylogenetic trees were used to calculate the interspecific phylogenetic distance (PDist) using the function *cophenetic* in the R package *stats* (R Core Team 2022). FDist and PDist were then integrated to generate a functional–phylogenetic distance matrix (FPDist) (Cadotte et al. 2013) using the function *funcy.phylo.dist* in the R package *pez* (Figure S1). FPDist non‐linearly combines FDist and PDist by assigning weights to both, whilst also accounting for whether the traits converge or diverge in the phylogenetic tree. Traits that have not been measured but are closely related to the phylogenetic tree are reflected in FPDist by weighted PDist (Cadotte et al. 2013). FPDist measures the weight of PDist based on the parameter *a*. Value *a* = 0 indicates that the interspecific distance is only measured by FDist, whereas *a* = 1 indicates that the interspecific distance is only measured by PDist, and when *a* lies between 0 and 1, FPDist is measured by both FDist and PDist.

### 2.6 Statistical Analyses

To infer the mechanism of community assembly, we adopted the method proposed by Si et al. (2017). We constructed a null model based on frequency, which maintains species occurrence frequency and randomizes community data matrix abundances within species (Table S4). The “frequency”‐based null model algorithm is sufficient for the adequate control of statistical errors (Miller et al. 2017). The model was run 999 times to generate 999 random clusters using the function *randomizeMatrix* in the R package *picante* (Kembel et al. 2010). Thereafter, we calculated the mean functional–phylogenetic pairwise distance (MFPD) and standardized effect size of MFPD (SES.MFPD) using the function *ses.mpd* in the R package *picante* (Kembel et al. 2010). Similar to the mean pairwise distance (MPD) (Webb et al. 2002), MFPD denotes the average distance between two species within a community after integrating FDist and PDist. By comparing observed MFPD values with the 999 random values to obtain SES.MFPD, it is possible to infer whether the structure of a community's functional–phylogenetic structure differs from random expectations. A negative SES.MFPD is taken to be indicative of a community that is functionally and phylogenetically clustered (clustering), whereas a positive SES.MFPD indicates that a community is functionally and phylogenetically dispersed (over‐dispersion) (Webb et al. 2002). Finally, to measure the strength of interspecific competition in the community, *a* was varied from 0 to 1 based on MPD to calculate the functional–phylogenetic evenness (FPDve) of birds along each surveyed reach using the function *dbFD* in the R package *FD* (Laliberté and Legendre 2010; Laliberté et al. 2014). Similar to functional evenness (FDve), FPDve is based on the minimum spanning tree linking all species in the multi‐dimensional functional space, and quantifies the regularity with which species abundance is distributed (Villéger et al. 2008). Some scholars believe that functional divergence (FDiv) reflects the degree of niche differentiation and resource competition among organisms within a community, with a higher FDiv value indicating stronger niche complementarity and weaker competition among the organisms within a community. However, FDiv reflects the distance of different species from the center of gravity of the convex hull of the functional space (Villéger et al. 2008), rather than the niche differentiation between two species, which is clearly inconsistent with the limiting similarity theory. Therefore, with reference to the methods proposed by Kraft and Ackerly (2010) and Zhang et al. (2020), we used the standard deviation of the nearest distances along a single direction (SDNNr) as a measure of limiting similarity, and the definition of FPDve was considered similar to SDNNr, which enabled us to adopt FPDve as the interspecific competition intensity (COMP) for the subsequent multiple linear regression analysis of SES.MFPD.

To determine the optimal value of *a* (i.e., the weight of PD), we varied *a* from 0 to 1 to establish the multiple linear regression model of SES.MFPD using the environmental variables (TEMP, PREC, NDVI, NPP, HII, and ALT) and competition intensity (COMP), and estimated the model's goodness‐of‐fit (adjusted *R* ^2^ value). When the model's adjusted *R* ^2^ reaches a maximum, this implies that the environmental variables and competition intensity can best explain the community function–phylogenetic structure, at which point, the value of *a* corresponding to the adjusted R^2^ value is the optimal value.

After determining the optimal value of *a*, to identify the main driver of community assembly (habitat filtering or interspecific competition), we re‐established the multiple linear regression model of SES.MFPD incorporating environmental variables (TEMP, PREC, NDVI, NPP, HII, and ALT) and competition intensity (COMP). The correlations between all variables were initially assessed using the function *ggpairs* in the R package *GGally* (Schloerke et al. 2021), and collinearity variables were removed. This was followed by multiple linear regression modeling. Variables were selected based on the significance of the regression coefficients, and the relative contributions of the significant variables to R^2^ were assessed using the function *calc.relimp* in the R package *relaimpo* (Grömping 2006). Given that the neutral theory proposes that dispersal limitation can also determine the patterns of community biodiversity (Hubbell 2001), we calculated the distance between river reaches using the function *distm* in the R package *geosphere* (Hijmans 2021), and assessed the spatial autocorrelation between SES.MFPD using the function *Moran.I* in the R package *ape* (Legendre and Legendre 2012; Paradis and Schliep 2019).

To determine whether the phylogenetic signal in traits related to community assembly is significant and whether observations of traits related to community assembly patterns match, we constructed a null model by maintaining species occurrence frequency and randomizing community data matrix abundances within species, performing 1000 iterations to generate 1000 random communities using the function *randomizeMatrix* in the R package *picante* (Kembel et al. 2010). In order to calculate MFPD for 1000 random communities and actual communities under different values of *a*, we varied *a* from 0 to 1 using the function *ses.mpd* in the R package *picante* (Kembel et al. 2010). To visualize whether the phylogenetic signal was significant and whether the observation of traits matched the community assembly patterns, the MFPDs of the 1000 random communities and actual communities were plotted graphically, with MFPD as the *y*‐axis and values of *a* along the *x*‐axis. All the aforementioned statistical analyses were performed using R 4.2.0 (R Core Team 2022).

## 3 Results

During the surveys conducted in this study, we recorded a total of 105 species of birds along the nine surveyed reaches for the three major reivers, with an average of 38 species per reach, with a standard deviation of 12, ranging from 23 to 55. Under different values of *a*, the maximum adjusted R^2^ for the multiple linear regression model of SES.MFPD with environmental variables (temperature, precipitation, normalized difference vegetation index, net primary productivity, human influence index, and altitude) and interspecific competition of the bird communities was 0.9999909, at which point, *a* took the value of 0.17 (Figure S2).

The mean value of SES.MFPD for the bird communities was −0.52 (standard deviation =1.56, ranging from −2.45 to 1.62; Table S4). Assessment of spatial autocorrelation of the distance between reaches with SES.MFPD indicated that there was no spatial autocorrelation (*p* = 0.71), thereby indicating that the distance effect did not affect community structure and there was no limit to dispersal.

**FIGURE 2:**
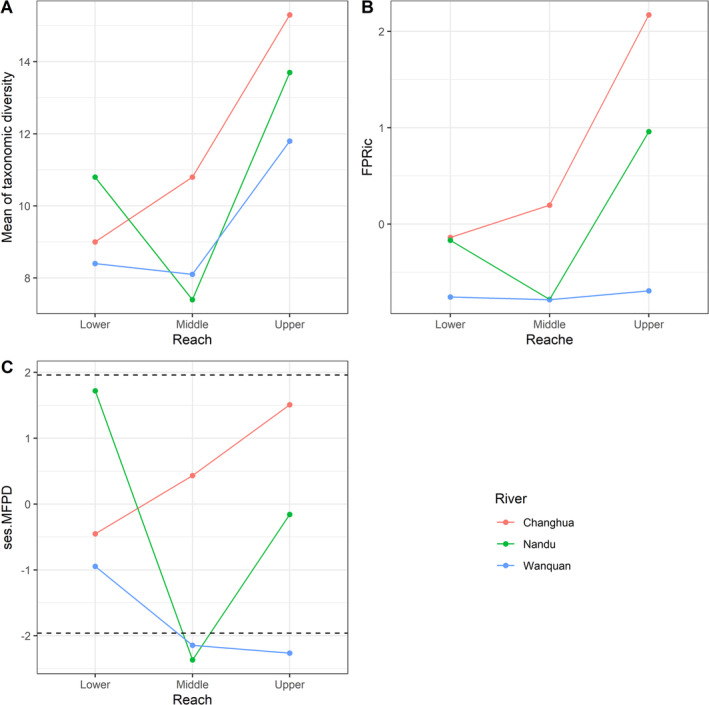
Mean taxonomic diversity calculated based on 10 transects established along different reaches of the three major rivers (A); functional–phylogenetic diversity of the different reaches determined using the *dbFD* function in the R package *FD* (B); standardized effect size of MFPD for each reach (C). The dashed line represents significant values (±1.98). “c” = the Changhua River, “n” = the Nandu River, “w” = the Wanquan River; “d” = downstream, “m” = midstream; “u” = upstream.

Given the aforementioned two points, we concluded that the functional‐phylogenetic structure of bird communities along the Nandu River, Changhua River and Wanquan Rivers is dominated by clustering. However, different reaches of these rivers were found to be characterized by different community functional‐phylogenetic structures. More specifically, the communities along the upper reaches of the Changhua River and the lower reaches of the Nandu River were found to be over‐dispersed, whereas those along the lower, middle, and upper reaches of the Wanquan River, and the middle reaches of the Nandu River showed clustering, and the other reaches showed a random pattern (Figure 2).

Correlation analyses for all variables revealed that temperature was strongly correlated with altitude, temperature, and normalized difference vegetation index (Spearman correlation analysis, correlation = −0.95 and *p* < 0.001 between temperature and altitude, correlation = −0.72 and *p* < 0.05 between temperature and normalized difference vegetation index) (Figure S3). On the basis of these finding, we re‐established the model after removing temperature. By manually removing non‐significant independent variables, we finally constructed multiple linear regression model of SES.MFPD incorporating altitude and human influence index (adjusted *R* ^2^ = 0.8734, *p* < 0.05 for altitude and *p* < 0.001 for human influence index), which revealed altitude and human influence index to be the main factors driving the functional–phylogenetic patterns of bird communities. Furthermore, we established that the contribution of human influence index to the adjusted *R* ^2^ (66.4%) was greater than that of altitude (24.1%) (Figure S4).

**FIGURE 3:**
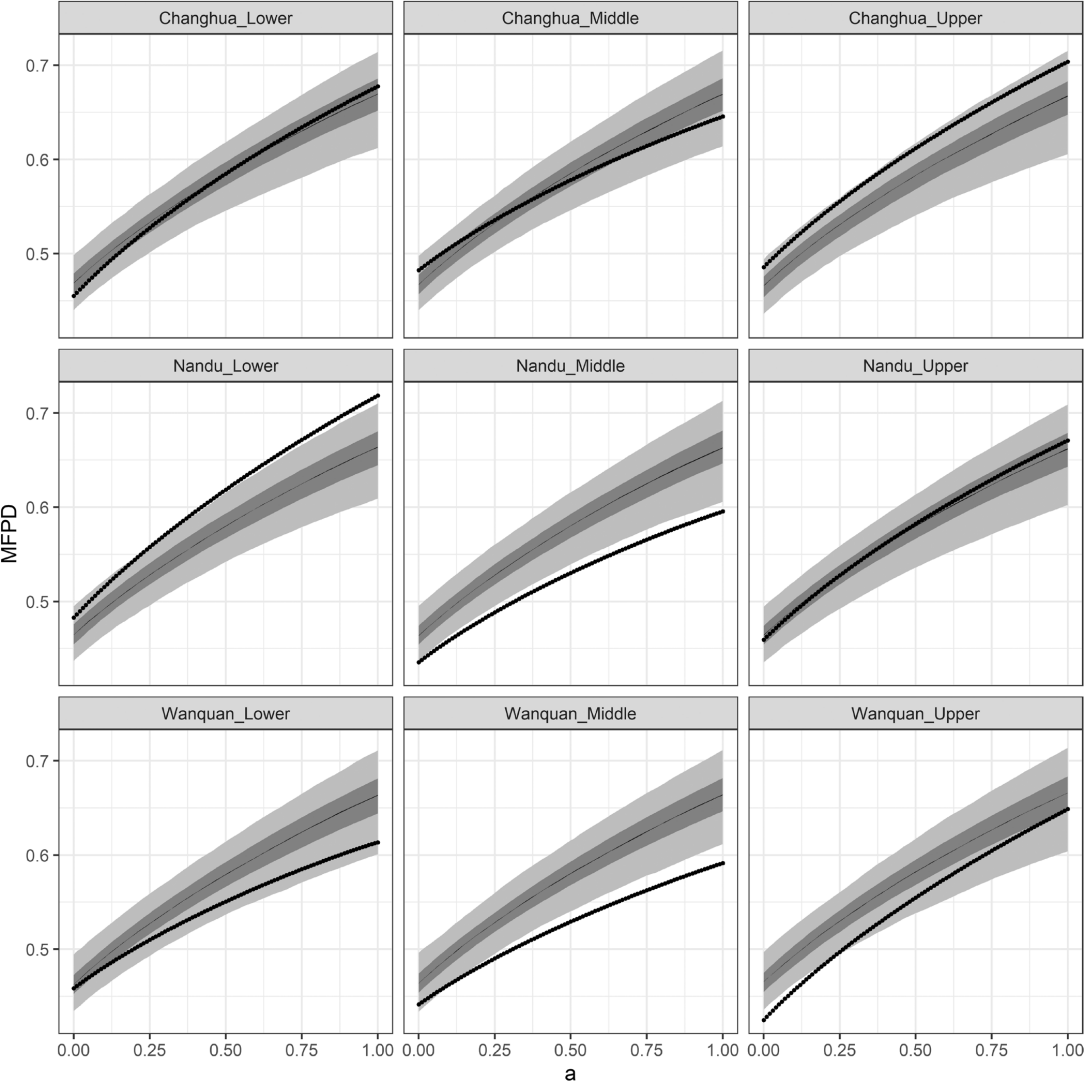
Mean pairwise functional–phylogenetic distances across the full range of the phylogenetic‐weighting parameter “*a*” for nine flow regimes. Shaded regions represent the null distribution assigned to the community‐specific richness (light gray, 0.025–0.975 = 95%, medium gray, 0.25–0.75 = 50%, solid line, 0.495–0.505 = 1%). The dotted lines show the observed mean pairwise distances. “c” = the Changhua River, “n” = the Nandu River, “w” = the Wanquan River; “d” = downstream, “m” = midstream; “u” = upstream.

Finally, our comparative analysis of the actual and predicted MFPD values under different values of *a* (i.e., different contributions of FDist and PDist to FPDist) indicated that, with the exception of for lower reaches of Changhua River, and upper reaches of Nandu River, which did not show significant community dispersion or clustering, all other reaches were characterized by community clustering or dispersion with the changes in *a*. More specifically, as shown in Figure 3, the curve of upper reaches of Changhua River was roughly parallel to the shaded area, indicating that the phylogenetic signals of traits associated with community assembly were signals and that the observation of traits matched the community assembly patterns. When values of *a* were small, middle reaches of Changhua River and upper reaches of Wanquan River showed dispersion or clustering, although shifted to random pattern as the values of *a* increased (i.e., as the contribution of PDist to FPDist increased), thereby indicating that the observation of traits matched community assembly patterns, although the phylogenetic signals of traits related to community assembly were not signals. With regards to the remaining four reaches (lower reaches of Nandu River and Wanquan River, middle reaches of Nandu River and Wanquan River), although the phylogenetic signals of traits related to community assembly were signals, the observation of traits related to community assembly did not match community assembly patterns, which is shown in Figure 3 as a shift in the community from a random pattern to dispersion or clustering with an increase int. the value of *a*.

## 4 Discussion

Our investigation has revealed that avian communities within the three major river basins of Hainan exhibit a subtle pattern of clustering. There are significant disparities in bird diversity and community assembly patterns across different river reaches, which are linked to deterministic processes of niche theory, primarily driven by altitude and the intensity of human impact. It is noteworthy that in areas with a higher degree of human influence, bird community assembly manifests a more over‐dispersion. We surmise that this may be due to anthropogenic disturbances within the river reaches leading to increased heterogeneity in land use patterns, thereby providing a greater array of niche spaces and resulting in the over‐dispersion of avian community structures. Concurrently, the inference of community assembly patterns varies significantly with the alteration of weights assigned to functional distance (FDist) and phylogenetic distance (PDist), denoted by the parameter *a*. By integrating functional and phylogenetic diversity, we can deduce whether the traits in question exhibit significant phylogenetic signals and whether they are correlated with community assembly, thus mitigating misinterpretations of community assembly patterns.

The convergence in functional and phylogenetic structures within communities may stem from a variety of ecological processes, including environmental filtering (Kraft et al. 2015), competitive dominance (Mayfield and Levine 2010), plant‐pollinator interactions and facilitation (Cavender‐Bares et al. 2009). Previous studies have seldom delved into the mechanisms of avian community assembly in riverine ecosystems, with only Royan et al. (2016) utilizing probabilistic models and joint species distribution models to reveal that environmental filtering, rather than species interactions, shapes the structure of avian communities along rivers. Our multivariate regression analysis indicated that habitat filtering played a predominant role in community assembly. River ecosystems are inherently disturbance‐prone, with fluctuations in river flow often altering the ecological structure (Junk et al. 1989), leading to spatial and temporal heterogeneity in foraging and breeding habitats for birds, ultimately resulting in biota turnover (Ward et al. 2002). Hydraulic facilities associated with rivers, such as canalization and dams, can to some extent lead to the degradation of river ecosystems, which is detrimental to the survival of certain bird species (Souza et al. 2019). Consequently, habitat may significantly influence the assembly of avian communities.

Our multivariate regression analysis indicated that altitude and the intensity of human impact are the primary drivers in the assembly of avian communities within the riverine ecosystems. Variations in altitude lead to significant changes in climate and biological factors along topographies and landscapes, impeding the dispersal of species with specific physical capacities and resulting in increased differentiation among species within communities (Basham et al. 2019). Consequently, our findings suggest that an increase in altitude leads to divergence in the functional‐phylogenetic structure of avian communities along rivers. However, considering the altitude variation in our study was only about 300 m, the explanatory power of altitude relative to human disturbance intensity is relatively low. Contrary to intuition, the structure of avian communities diverges with increasing intensity of human impact, indicating that anthropogenic disturbances promote the coexistence of birds with different ecological niches. Previous studies have shown that in riparian ecosystems with moderate disturbance levels, taxonomic diversity, functional richness, and functional diversity are maximized (Biswas and Mallik 2010). Therefore, moderate human disturbances within the three major river basins may facilitate species coexistence. Since different species within a community may respond variably to abiotic conditions, abiotic heterogeneity can lead to the coexistence of species with substantial differences (Chesson 2000; Snyder and Chesson 2004; Kraft et al. 2015). Anthropogenic disturbances may generate heterogeneous land use types, providing more niche space and leading to the divergence of avian communities. For instance, in the lower reaches of the Nandu River, habitats were predominantly cropland, and avian communities exhibit over‐dispersion. In contrast, other downstream sections (lower reaches of Changhua River and Wanquan River) with habitats mainly consisting of countryside and rubber plantations show clustering. Although cropland, countryside, and plantations are all anthropogenic habitats, cropland serves as foraging sites for many waders, egrets, and raptors (Gunathilaka 2019; Acosta et al. 2010). Some studies also indicate that cropland has higher bird diversity than oil palm and rubber plantations (Li et al. 2013; Azman et al. 2011). Diversified agricultural systems can also buffer the loss of avian phylogenetic diversity in the context of intensive agricultural systems (Frishkoff et al. 2014).

Finally, we explored the merits of integrating functional and phylogenetic diversity in elucidating actual community assembly mechanisms. Firstly, phylogenetic diversity‐based methods necessitate that traits exhibit phylogenetic signals (Lean and Maclaurin 2016). However, a functional‐phylogenetic approach does not require prior verification of phylogenetic signals for traits. When traits with weak phylogenetic signals reflect community assembly mechanisms (e.g., middle reaches of Changhua River and upper reaches of Wanquan River), as the proportion of PDist (denoted by the magnitude of *a*) increases, the SES.MFPD will deviate towards a random community pattern. This is because PDist cannot accurately represent the true ecological niche differences among species within the community. Additionally, the functional‐phylogenetic approach can also reveal whether the selected traits genuinely drive community assembly. When selected traits have significant phylogenetic signals but fail to reflect community assembly mechanisms (e.g., middle and lower reaches of Nandu River and Wanquan River), as the weight of PDist (the magnitude of *a*) increases, the SES.MFPD will shift from a random community pattern towards clustering or over‐dispersion. This is because PDist better reflects niche differentiation among species within the community compared to FDist. By employing different weights of FDist and PDist, and regressing SES.MFPD against environmental variables and interspecific competition intensity, the optimal value of *a* that best explains the model represents the ideal weighting for FDist and PDist. If one were to consider only functional diversity (*a* = 0) and phylogenetic diversity (*a* = 1), the inferred community structure patterns would yield significantly divergent conclusions.

## Author Contributions

**Sidan Lin:** data curation (lead), formal analysis (lead), investigation (lead), methodology (equal), writing – original draft (equal). **Wei Liang:** conceptualization (lead), funding acquisition (lead), supervision (lead), validation (equal), writing – review and editing (lead).

## Ethics Statement

The experiments comply with the current laws of China. No special permit was required for this study as it was not involved in animal or plant collection.

## Conflicts of Interest

The authors declare no conflicts of interest.

## Supporting information


Figure S1.

Figure S2.

Figure S3.

Figure S4.

Data S1.

## Data Availability

Data used for this study are provided as Supporting Information (Figures [Link], [Link], [Link], [Link] and Tables S1–S4), and can be found online at https://figshare.com/s/cecd93ef331ec13b5b60 (doi: 10.6084/m9.figshare.27887382).
