# Supplementary figures and images for: Integrating Functional and Phylogenetic Diversity to Assess Bird Community Assembly Along the Major Rivers of Hainan Island, South China
Source: Ecol Evol. 2025 Feb 10;15(2):e70962. doi: 10.1002/ece3.70962 (PMC11808277; doi:10.1002/ece3.70962)

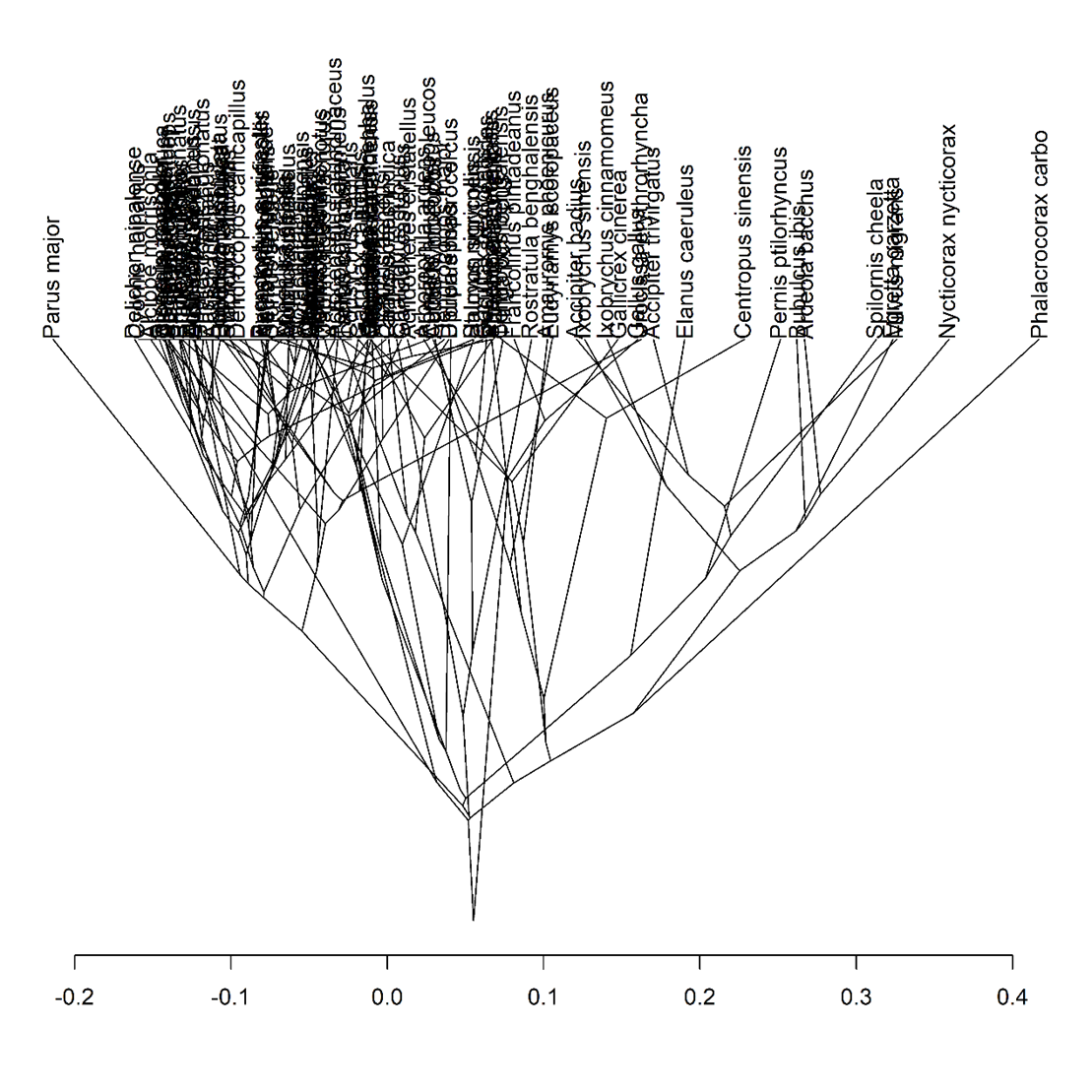

Supplement: Supplementary file 1 — Figure S1. [file ECE3-15-e70962-s004.tif]

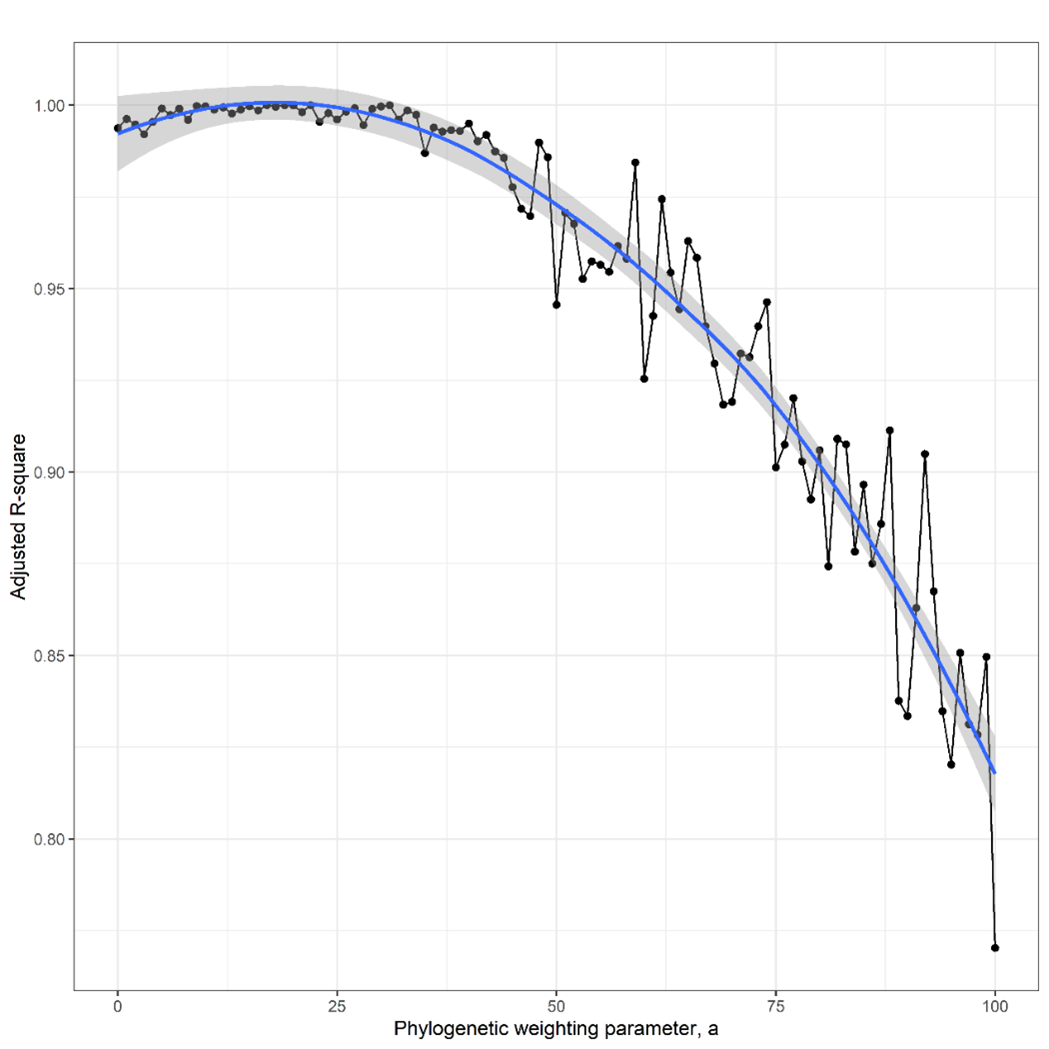

Supplement: Supplementary file 2 — Figure S2. [file ECE3-15-e70962-s002.tif]

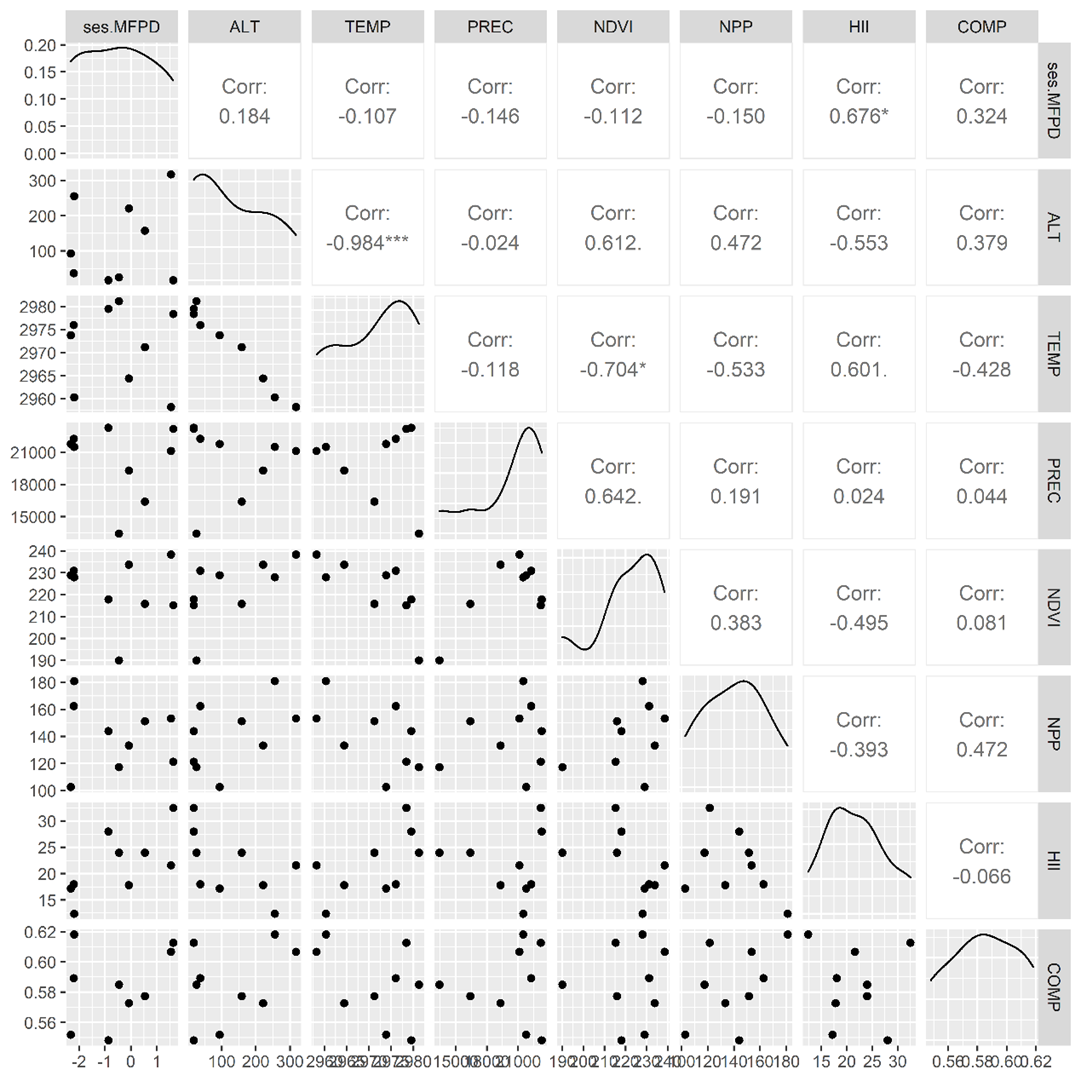

Supplement: Supplementary file 3 — Figure S3. [file ECE3-15-e70962-s003.tif]

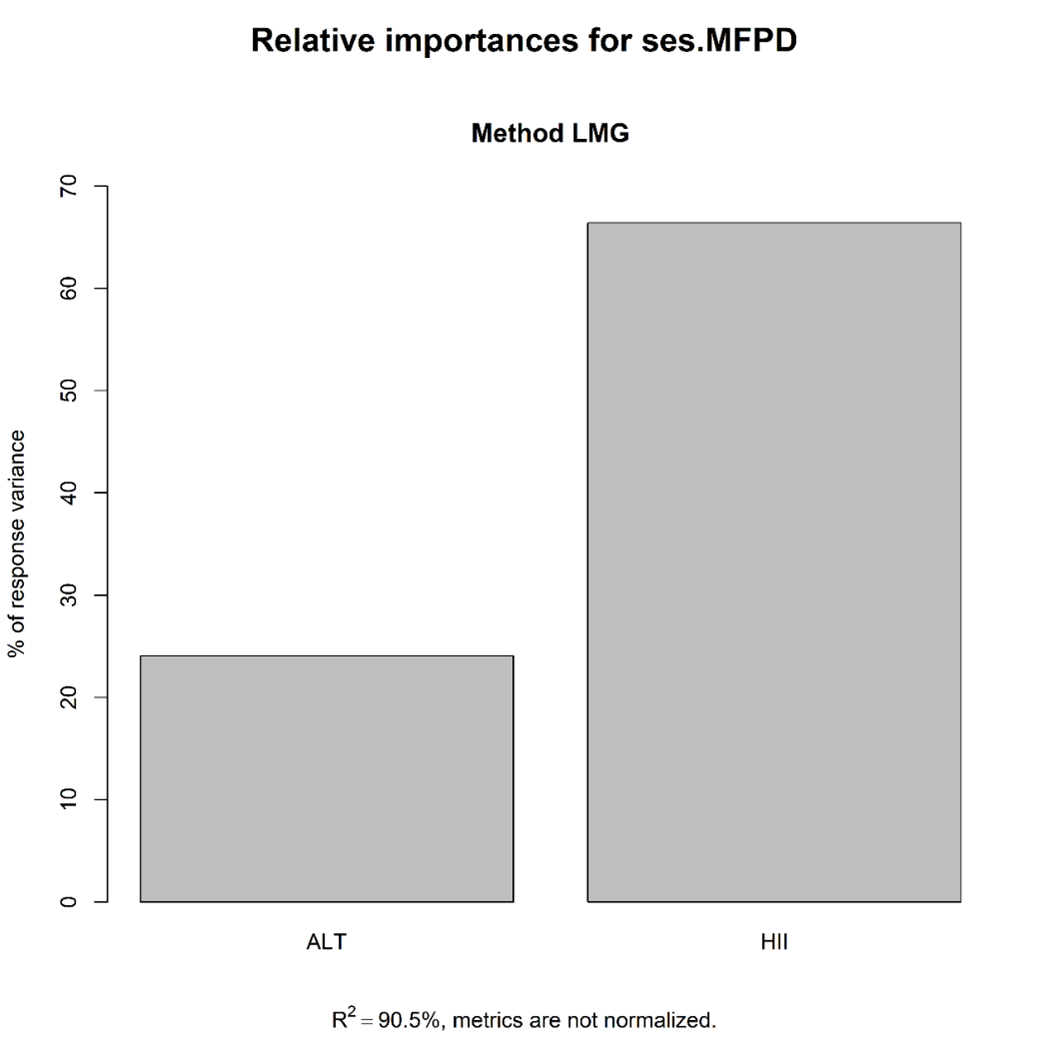

Supplement: Supplementary file 4 — Figure S4. [file ECE3-15-e70962-s001.tif]
