# Supplementary material for: Integrating Functional and Phylogenetic Diversity to Assess Bird Community Assembly Along the Major Rivers of Hainan Island, South China
Source: Ecol Evol. 2025 Feb 10;15(2):e70962. doi: 10.1002/ece3.70962 (PMC11808277; doi:10.1002/ece3.70962)
Supplement: Supplementary file 5 — Data S1. [file ECE3-15-e70962-s005.docx]

**Supplementary material**

**Figure S1**. Traitgram of birds in study rivers. Traitgrams combine phylogenetic trees and functional trait space. Functional phylogenetic distances in traitgram space are calculated as follows: $FPDist={(a{PDist}^{p}+\left( 1-a \right){FDist}^{p})}^{1/p}$(p = 2), which includes trait convergence and divergence into phylogenetic distances. X-axis represents the first axis of the principal coordinates analysis driven on the measured traits.

**Figure S2**. Adjusted R-squared values of the regression between ses.MFPD and environmental variables across the range of a-values.

**Figure S3**. Correlation test between environmental variables and ese.MFPD. The lower left triangle values and diagonal values represents the distribution of the data, the upper right triangle represents the correlation coefficient and its significance using Spearman’s correlation test. “*” = “*P* < 0.05”, “**” = “*P* < 0.01”, “***” = “*P* < 0.001”.

**Figure S4**. Relative importance of significant environmental variables for ses.MFPD. The total R-square is 90.5%, relative importance of ALT is 24.1% and HII is 66.4%.


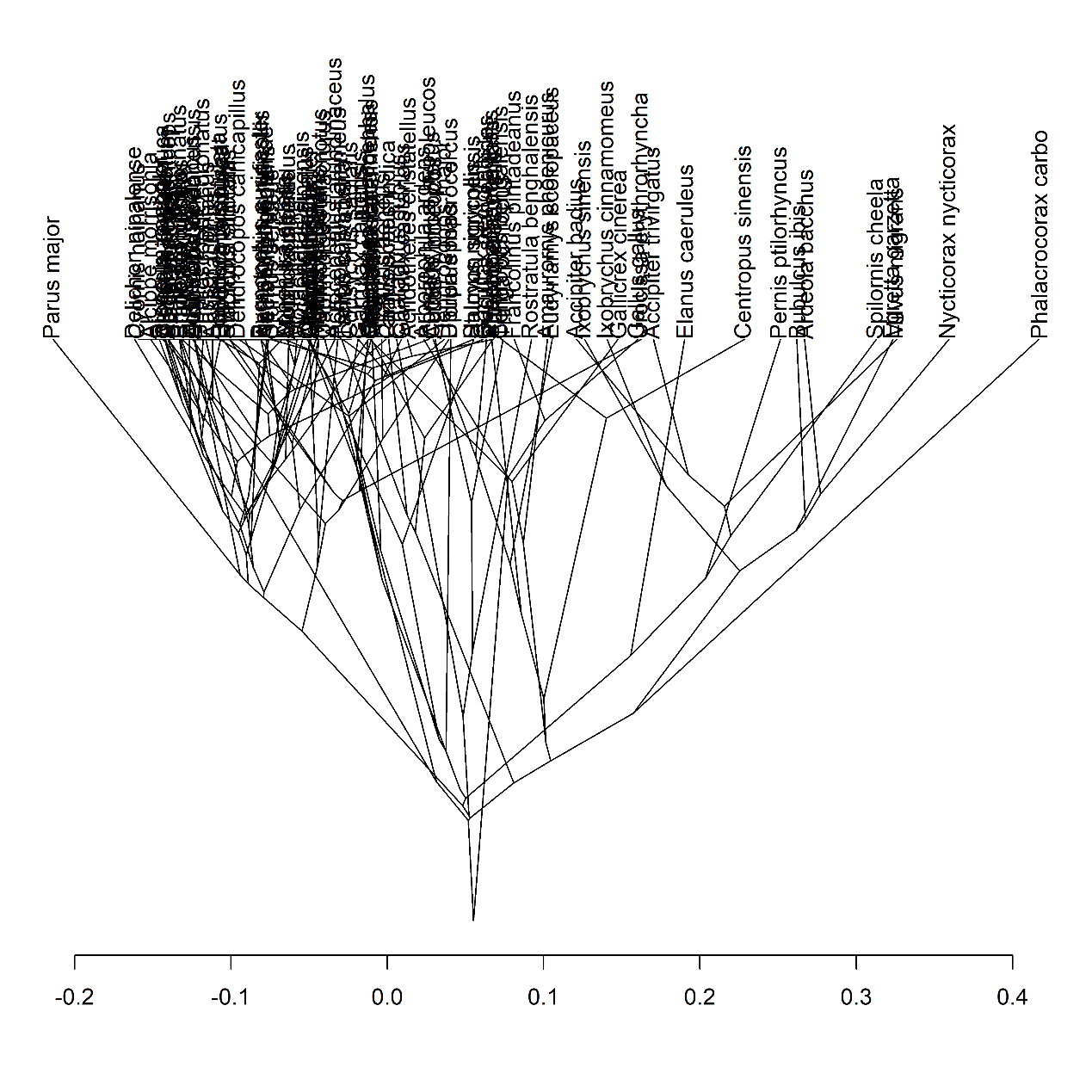


**Fig. S1**


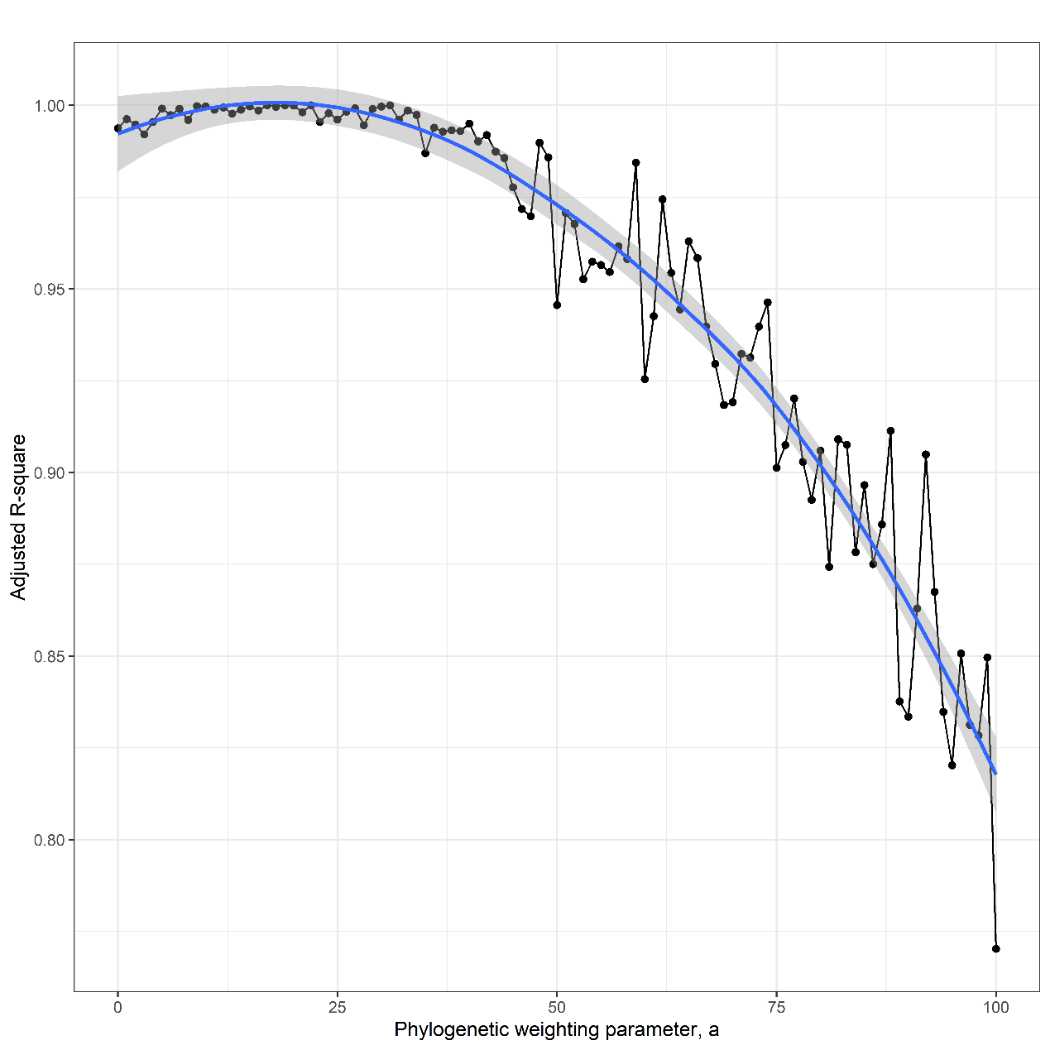


**Fig. S2**


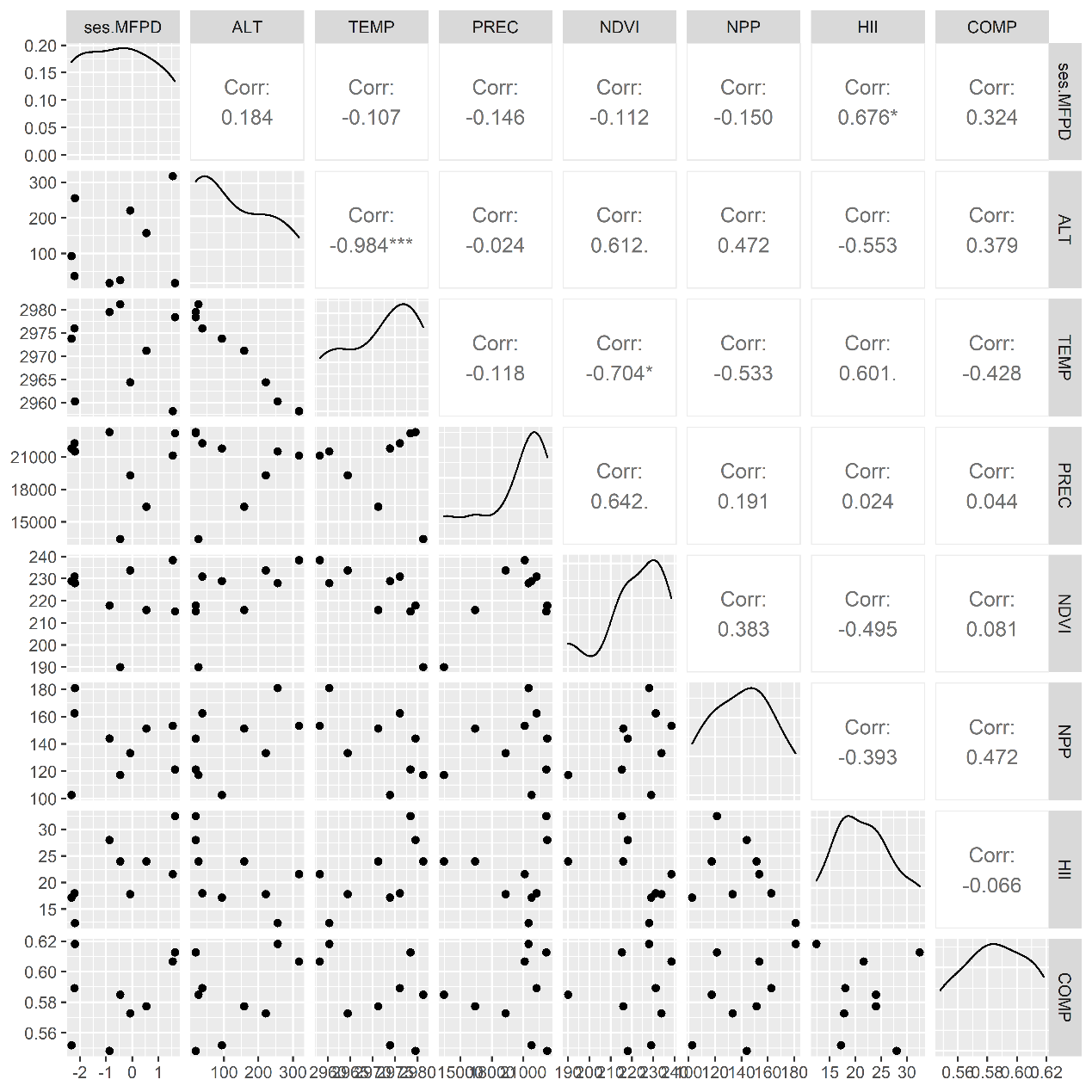


**Fig. S3**


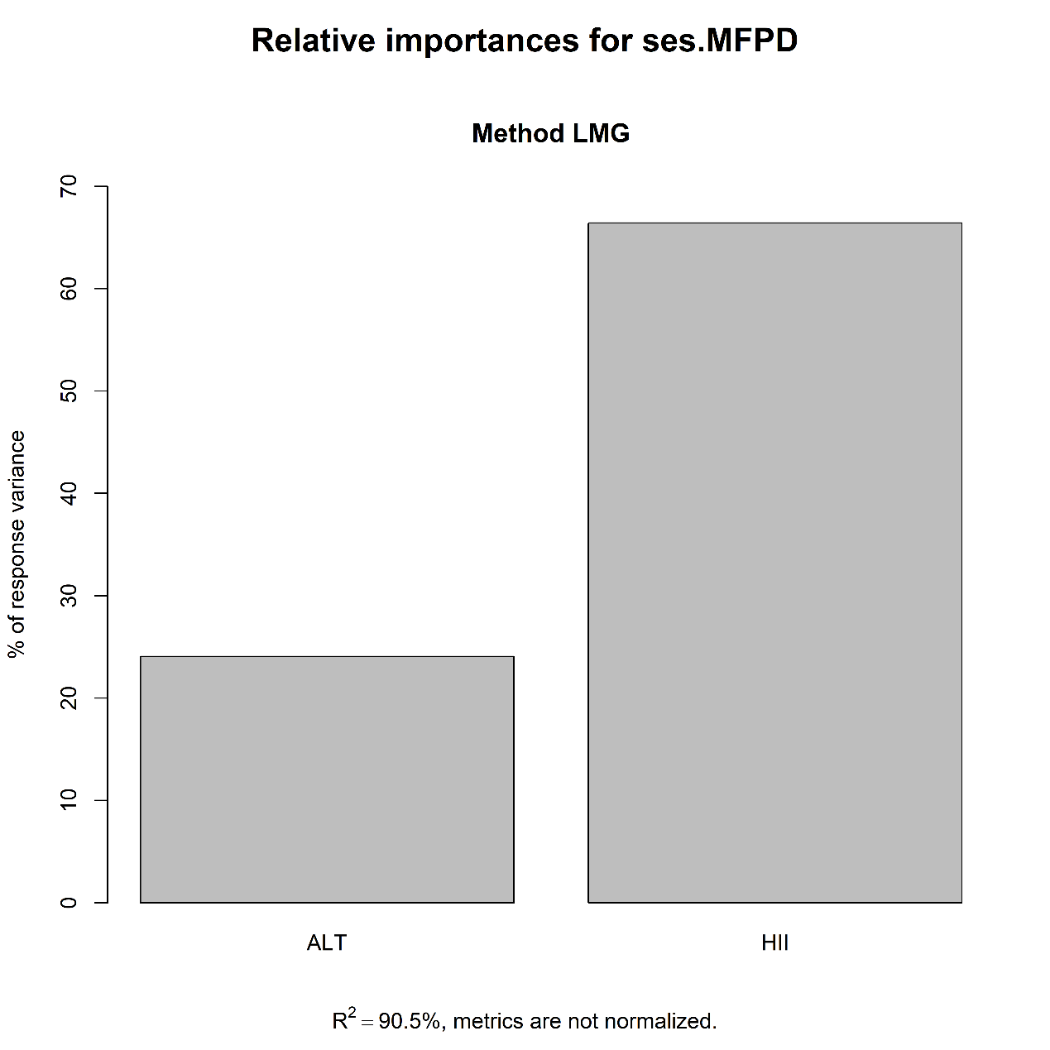


**Fig. S4**

**Table S1**. Occupancy data of birds in study flow regimes by species (rows) across river site (columns). “c” means “Changhua River”, “n” refers to “Nandu River”, “w” refers to “Wanquan River”.

| Species/River.site | c_d | c_m | c_u | n_d | n_m | n_u | w_d | w_m | w_u |
| --- | --- | --- | --- | --- | --- | --- | --- | --- | --- |
| *Accipiter_badius* | 1 | 1 | 1 | 0 | 1 | 0 | 0 | 0 | 1 |
| *Accipiter_trivirgatus* | 0 | 0 | 1 | 0 | 0 | 0 | 0 | 0 | 0 |
| *Acridotheres_cristatellus* | 1 | 1 | 1 | 1 | 0 | 0 | 1 | 1 | 1 |
| *Acridotheres_tristis* | 0 | 1 | 0 | 1 | 0 | 0 | 1 | 0 | 0 |
| *Acrocephalus_arundinaceus* | 1 | 0 | 0 | 0 | 0 | 0 | 0 | 0 | 0 |
| *Actitis_hypoleucos* | 0 | 0 | 0 | 0 | 0 | 1 | 0 | 0 | 0 |
| *Aethopyga_christinae* | 1 | 1 | 1 | 0 | 1 | 1 | 1 | 1 | 1 |
| *Alauda_gulgula* | 1 | 0 | 0 | 0 | 0 | 1 | 0 | 0 | 0 |
| *Alcedo_atthis* | 0 | 0 | 0 | 0 | 0 | 0 | 0 | 0 | 1 |
| *Alcippe_brunnea* | 0 | 1 | 1 | 0 | 1 | 0 | 0 | 0 | 1 |
| *Alcippe_morrisonia* | 0 | 0 | 0 | 0 | 0 | 1 | 0 | 0 | 1 |
| *Alophoixus_pallidus* | 0 | 0 | 0 | 0 | 0 | 1 | 0 | 1 | 0 |
| *Amaurornis_phoenicurus* | 1 | 0 | 1 | 0 | 0 | 0 | 1 | 1 | 1 |
| *Anthus_hodgsoni* | 0 | 0 | 1 | 0 | 0 | 1 | 0 | 0 | 0 |
| *Anthus_richardi* | 0 | 0 | 0 | 0 | 0 | 1 | 0 | 0 | 0 |
| *Apus_nipalensis* | 1 | 1 | 1 | 1 | 0 | 1 | 0 | 0 | 0 |
| *Apus_pacificus* | 1 | 0 | 0 | 0 | 0 | 0 | 0 | 0 | 0 |
| *Arborophila_ardens* | 0 | 0 | 0 | 0 | 0 | 1 | 0 | 0 | 0 |
| *Ardeola_bacchus* | 1 | 0 | 1 | 1 | 0 | 1 | 1 | 1 | 1 |
| *Artamus_fuscus* | 0 | 1 | 1 | 1 | 0 | 1 | 0 | 0 | 0 |
| *Bubulcus_ibis* | 0 | 1 | 0 | 1 | 0 | 0 | 0 | 0 | 0 |
| *Caprimulgus_macrurus* | 0 | 0 | 1 | 0 | 0 | 1 | 0 | 0 | 0 |
| *Centropus_bengalensis* | 1 | 1 | 1 | 1 | 0 | 1 | 0 | 0 | 0 |
| *Centropus_sinensis* | 1 | 1 | 1 | 1 | 0 | 1 | 1 | 1 | 1 |
| *Chalcophaps_indica* | 0 | 0 | 0 | 0 | 0 | 0 | 0 | 0 | 1 |
| *Charadrius_dubius* | 1 | 0 | 0 | 0 | 0 | 1 | 0 | 0 | 0 |
| *Cisticola_juncidis* | 1 | 0 | 0 | 1 | 0 | 0 | 0 | 0 | 0 |
| *Copsychus_saularis* | 1 | 1 | 1 | 1 | 1 | 1 | 1 | 1 | 1 |
| *Cuculus_micropterus* | 0 | 0 | 0 | 1 | 1 | 1 | 1 | 0 | 1 |
| *Cuculus_sparverioides* | 0 | 1 | 1 | 0 | 0 | 1 | 0 | 0 | 1 |
| *Culicicapa_ceylonensis* | 0 | 0 | 0 | 0 | 0 | 0 | 0 | 0 | 1 |
| *Cyornis_hainanus* | 0 | 0 | 1 | 0 | 1 | 1 | 1 | 1 | 1 |
| *Delichon_nipalense* | 0 | 0 | 1 | 0 | 0 | 1 | 0 | 0 | 0 |
| *Dendrocopos_canicapillus* | 1 | 0 | 1 | 0 | 0 | 1 | 0 | 1 | 1 |
| *Dendrocopos_major* | 0 | 0 | 0 | 0 | 0 | 0 | 0 | 0 | 1 |
| *Dicaeum_concolor* | 0 | 0 | 1 | 1 | 0 | 0 | 1 | 1 | 1 |
| *Dicaeum_cruentatum* | 1 | 1 | 1 | 1 | 1 | 0 | 1 | 1 | 1 |
| *Dicrurus_aeneus* | 0 | 0 | 0 | 0 | 0 | 0 | 0 | 0 | 1 |
| *Dicrurus_macrocercus* | 1 | 0 | 0 | 1 | 0 | 0 | 0 | 0 | 0 |
| *Egretta_garzetta* | 0 | 1 | 1 | 1 | 1 | 1 | 0 | 0 | 0 |
| *Elanus_caeruleus* | 1 | 0 | 1 | 1 | 0 | 0 | 0 | 0 | 0 |
| *Emberiza_pusilla* | 0 | 0 | 0 | 0 | 0 | 1 | 0 | 0 | 0 |
| *Erpornis_zantholeuca* | 0 | 1 | 1 | 0 | 0 | 1 | 0 | 1 | 1 |
| *Eudynamys_scolopaceus* | 0 | 0 | 1 | 1 | 0 | 0 | 0 | 0 | 0 |
| *Francolinus_pintadeanus* | 1 | 1 | 1 | 1 | 0 | 1 | 1 | 0 | 0 |
| *Gallicrex_cinerea* | 0 | 0 | 0 | 1 | 0 | 0 | 0 | 0 | 0 |
| *Gallus_gallus* | 0 | 0 | 1 | 0 | 0 | 0 | 0 | 0 | 0 |
| *Garrulax_canorus* | 0 | 0 | 0 | 0 | 0 | 1 | 0 | 0 | 0 |
| *Garrulax_castanotis* | 0 | 1 | 0 | 0 | 0 | 0 | 0 | 0 | 0 |
| *Garrulax_chinensis* | 1 | 1 | 1 | 1 | 1 | 1 | 0 | 0 | 0 |
| *Garrulax_pectoralis* | 0 | 1 | 0 | 0 | 0 | 0 | 0 | 0 | 0 |
| *Glaucidium_brodiei* | 0 | 0 | 0 | 0 | 0 | 0 | 0 | 0 | 1 |
| *Glaucidium_cuculoides* | 0 | 0 | 1 | 0 | 1 | 1 | 0 | 0 | 0 |
| *Halcyon_smyrnensis* | 0 | 1 | 1 | 1 | 1 | 1 | 1 | 1 | 1 |
| *Hemixos_castanonotus* | 0 | 0 | 1 | 0 | 0 | 1 | 0 | 0 | 1 |
| *Hirundo_rustica* | 1 | 1 | 1 | 1 | 1 | 1 | 1 | 1 | 1 |
| *Hypothymis_azurea* | 0 | 1 | 0 | 1 | 1 | 0 | 0 | 1 | 1 |
| *Hypsipetes_leucocephalus* | 0 | 1 | 1 | 0 | 0 | 0 | 0 | 0 | 0 |
| *Ixobrychus_cinnamomeus* | 0 | 0 | 0 | 1 | 0 | 0 | 0 | 0 | 0 |
| *Ixobrychus_sinensis* | 0 | 1 | 0 | 1 | 0 | 0 | 0 | 0 | 0 |
| *Lanius_cristatus* | 1 | 1 | 0 | 0 | 0 | 0 | 0 | 0 | 1 |
| *Lanius_schach* | 1 | 1 | 1 | 1 | 0 | 1 | 1 | 1 | 1 |
| *Lonchura_punctulata* | 1 | 1 | 1 | 0 | 0 | 1 | 1 | 0 | 0 |
| *Lonchura_striata* | 1 | 1 | 1 | 0 | 0 | 0 | 1 | 1 | 1 |
| *Megalaima_faber* | 1 | 1 | 1 | 0 | 0 | 1 | 0 | 0 | 1 |
| *Milvus_migrans* | 0 | 0 | 1 | 0 | 0 | 1 | 0 | 0 | 0 |
| *Monticola_gularis* | 0 | 0 | 0 | 0 | 0 | 1 | 0 | 0 | 0 |
| *Motacilla_alba* | 1 | 0 | 1 | 0 | 0 | 1 | 0 | 0 | 1 |
| *Motacilla_cinerea* | 0 | 0 | 1 | 0 | 0 | 1 | 0 | 0 | 0 |
| *Nectarinia_jugularis* | 1 | 1 | 1 | 0 | 1 | 1 | 1 | 1 | 1 |
| *Nycticorax_nycticorax* | 0 | 1 | 0 | 0 | 0 | 0 | 0 | 0 | 0 |
| *Otus_lettia* | 1 | 0 | 0 | 0 | 0 | 1 | 0 | 0 | 0 |
| *Otus_spilocephalus* | 0 | 0 | 1 | 0 | 0 | 0 | 0 | 0 | 0 |
| *Parus_major* | 1 | 1 | 1 | 1 | 1 | 1 | 1 | 1 | 1 |
| *Passer_montanus* | 1 | 0 | 0 | 0 | 0 | 0 | 1 | 0 | 0 |
| *Pericrocotus_flammeus* | 0 | 1 | 1 | 0 | 1 | 1 | 0 | 0 | 1 |
| *Pernis_ptilorhyncus* | 0 | 0 | 1 | 0 | 0 | 0 | 0 | 0 | 0 |
| *Phaenicophaeus_tristis* | 0 | 0 | 0 | 1 | 0 | 0 | 0 | 0 | 0 |
| *Phalacrocorax_carbo* | 0 | 1 | 0 | 0 | 0 | 0 | 0 | 0 | 0 |
| *Phylloscopus_coronatus* | 0 | 1 | 0 | 0 | 0 | 0 | 0 | 0 | 0 |
| *Phylloscopus_fuscatus* | 0 | 0 | 1 | 0 | 0 | 1 | 0 | 0 | 0 |
| *Phylloscopus_inornatus* | 1 | 0 | 0 | 0 | 0 | 1 | 0 | 0 | 0 |
| *Pomatorhinus_hypoleucos* | 0 | 1 | 0 | 0 | 1 | 0 | 0 | 0 | 0 |
| *Pomatorhinus_ruficollis* | 0 | 1 | 0 | 0 | 1 | 1 | 0 | 1 | 1 |
| *Porzana_fusca* | 0 | 0 | 0 | 1 | 0 | 0 | 0 | 0 | 0 |
| *Prinia_flaviventris* | 1 | 1 | 1 | 1 | 0 | 1 | 1 | 1 | 1 |
| *Prinia_inornata* | 1 | 0 | 0 | 0 | 0 | 1 | 0 | 0 | 0 |
| *Pycnonotus_aurigaster* | 0 | 0 | 0 | 1 | 0 | 0 | 0 | 0 | 0 |
| *Pycnonotus_sinensis* | 1 | 1 | 1 | 1 | 1 | 1 | 1 | 1 | 1 |
| *Rhipidura_albicollis* | 0 | 0 | 0 | 0 | 1 | 0 | 0 | 0 | 0 |
| *Rostratula_benghalensis* | 1 | 0 | 0 | 1 | 0 | 0 | 0 | 0 | 0 |
| *Seicercus_castaniceps* | 0 | 1 | 0 | 0 | 0 | 0 | 0 | 0 | 0 |
| *Spilornis_cheela* | 0 | 1 | 1 | 0 | 0 | 1 | 0 | 0 | 0 |
| *Stachyris_ruficeps* | 0 | 1 | 1 | 0 | 1 | 1 | 0 | 1 | 1 |
| *Stachyris_striolata* | 0 | 0 | 0 | 0 | 0 | 1 | 0 | 0 | 1 |
| *Stigmatopelia_chinensis* | 1 | 1 | 1 | 1 | 1 | 1 | 1 | 1 | 1 |
| *Streptopelia_orientalis* | 0 | 0 | 0 | 0 | 0 | 1 | 0 | 0 | 0 |
| *Sturnus_nigricollis* | 1 | 0 | 1 | 1 | 0 | 0 | 1 | 0 | 0 |
| *Sturnus_sericeus* | 0 | 0 | 0 | 1 | 0 | 0 | 0 | 0 | 0 |
| *Sturnus_sinensis* | 0 | 0 | 0 | 0 | 0 | 1 | 1 | 0 | 0 |
| *Treron_curvirostra* | 0 | 0 | 0 | 0 | 0 | 0 | 0 | 0 | 1 |
| *Tringa_ochropus* | 0 | 0 | 1 | 0 | 0 | 0 | 0 | 0 | 0 |
| *Upupa_epops* | 0 | 0 | 0 | 1 | 0 | 0 | 0 | 0 | 0 |
| *Urocissa_erythrorhyncha* | 1 | 0 | 0 | 0 | 0 | 0 | 0 | 0 | 0 |
| *Zosterops_japonicus* | 0 | 1 | 1 | 0 | 1 | 1 | 1 | 1 | 1 |

**Table S2**. Environmental data of the study rivers. “c” means “Changhua River”, “n” refers to “Nandu River”, “w” refers to “Wanquan River”. “d” refers to downstream, “m” refers to midstream, “u” refers to upstream. ALT = altitude, TEMP = mean annual air temperature, PREC = annual precipitation amount, NDVI = normalized difference vegetation index, NPP = net primary productivity, HII = human influence index.

| River | Site | latitude | longitude | ALT | TEMP | PREC | NDVI | NPP | HII |
| --- | --- | --- | --- | --- | --- | --- | --- | --- | --- |
| c | d | 19.2223708 | 108.922523 | 25.35 | 2981.2 | 13436.7 | 190 | 117.4 | 24 |
| c | m | 18.7932048 | 109.157413 | 158 | 2971.2 | 16410.2 | 215.8 | 151.4 | 24 |
| c | u | 19.0036301 | 109.652561 | 317.35 | 2958.2 | 21117.8 | 238.4 | 153.4 | 21.6 |
| n | d | 19.7651121 | 110.359025 | 17.45 | 2978.4 | 23169.1 | 215.2 | 121.3 | 32.5 |
| n | m | 19.5412623 | 109.944309 | 93.6 | 2973.8 | 21768.2 | 228.9 | 102.6 | 17.2 |
| n | u | 19.15181 | 109.456872 | 220.8 | 2964.4 | 19289.6 | 233.7 | 133.3 | 17.8 |
| w | d | 19.1999829 | 110.431464 | 17.2 | 2979.5 | 23263.5 | 217.9 | 144 | 28 |
| w | m | 19.0974953 | 110.272659 | 36.9 | 2976 | 22241.4 | 231 | 162.5 | 18 |
| w | u | 18.8786136 | 109.847868 | 255.45 | 2960.3 | 21487 | 228 | 181 | 12.4 |

**Table S3**. Trait data of birds in the study rivers.

| Species | Beak.Length_Culmen | Beak.Length_res | Beak.Width | Beak.Depth | Tarsus.Length | Wing.Length | Kipps.Distance | Secondary1 | Hand-Wing.Index | Tail.Length | Mass | Habitat | Trophic.Level | Trophic.Niche | Primary.Lifestyle | Nocturl | clutch.min | clutch.max | nest.closeORopen | nest.location |
| --- | --- | --- | --- | --- | --- | --- | --- | --- | --- | --- | --- | --- | --- | --- | --- | --- | --- | --- | --- | --- |
| Zosterops_japonicus | 13.4 | 7.6 | 2.8 | 2.9 | 17.1 | 56.8 | 11.3 | 45.5 | 19.9 | 41.6 | 11.3 | Forest | Omnivore | Omnivore | Insessorial | 0 | 3 | 4 | open | bush |
| Acridotheres_cristatellus | 27.1 | 15.7 | 6.1 | 8.1 | 38.1 | 134 | 32.1 | 101.5 | 24.1 | 81.2 | 116.3 | Grassland | Omnivore | Omnivore | Generalist | 0 | 4 | 5 | close | canopy |
| Erpornis_zantholeuca | 15.4 | 8.4 | 3.9 | 4.2 | 16.7 | 66.4 | 11.3 | 54.8 | 17.1 | 44 | 11.8 | Forest | Omnivore | Invertivore | Insessorial | 0 | 2 | 3 | open | bush |
| Alophoixus_pallidus | 20.3 | 12.1 | 4.6 | 7.4 | 20.5 | 102.6 | 15.6 | 87 | 15.2 | 104.2 | 46 | Forest | Herbivore | Frugivore | Insessorial | 0 | 2 | 4 | open | bush |
| Pycnonotus_aurigaster | 19.4 | 9.8 | 4.1 | 4.8 | 21.9 | 91.8 | 14 | 76.4 | 15.5 | 82.4 | 44.3 | Shrubland | Herbivore | Omnivore | Insessorial | 0 | 2 | 3 | open | bush |
| Monticola_gularis | 20.4 | 11.2 | 4.4 | 5.8 | 23.8 | 97 | 30.7 | 66.1 | 31.7 | 64.2 | 34.6 | Forest | Carnivore | Invertivore | Terrestrial | 0 | 4 | 8 | open | groud |
| Rhipidura_albicollis | 13.6 | 6.5 | 4 | 3.1 | 18.9 | 78.4 | 13.8 | 64.6 | 17.6 | 100.3 | 12.9 | Forest | Carnivore | Invertivore | Insessorial | 0 | 3 | 3 | open | bush |
| Motacilla_alba | 16.1 | 9.7 | 3.4 | 3.5 | 23.4 | 87 | 27.4 | 59.7 | 31.4 | 86.8 | 23.9 | Human Modified | Carnivore | Invertivore | Terrestrial | 0 | 5 | 6 | open | groud |
| Egretta_garzetta | 94.6 | 68 | 8.9 | 11.3 | 98.8 | 252.5 | 82.8 | 167.2 | 33.1 | 94.2 | 312 | Wetland | Carnivore | Aquatic predator | Terrestrial | 0 | 3 | 6 | open | canopy |
| Pycnonotus_sinensis | 17.9 | 8.6 | 4.4 | 4.9 | 21.6 | 86.7 | 17.8 | 68.1 | 20.8 | 82.1 | 34.2 | Human Modified | Herbivore | Frugivore | Insessorial | 0 | 4 | 4 | open | canopy |
| Halcyon_smyrnensis | 59.8 | 48.4 | 13.7 | 14.3 | 15 | 113.2 | 24.5 | 88.7 | 21.7 | 78.3 | 91.4 | Human Modified | Carnivore | Omnivore | Insessorial | 0 | 5 | 7 | close | rock-face |
| Amaurornis_phoenicurus | 37.1 | 18.6 | 4.4 | 8.8 | 50.8 | 147 | 33.7 | 111.5 | 23.2 | 57.6 | 180 | Wetland | Carnivore | Aquatic predator | Terrestrial | 0 | 4 | 8 | open | bush |
| Tringa_ochropus | 37.4 | 27.6 | 2.7 | 4.1 | 32.9 | 137.8 | 63.2 | 74.8 | 45.8 | 59.5 | 71.4 | Wetland | Carnivore | Aquatic predator | Terrestrial | 0 | 3 | 4 | open | groud |
| Lonchura_striata | 12 | 8.3 | 6.7 | 7.8 | 13.1 | 50.7 | 10.6 | 40.1 | 20.9 | 39.8 | 12.3 | Shrubland | Herbivore | Granivore | Insessorial | 0 | 4 | 6 | open | canopy |
| Apus_pacificus | 8 | 4.6 | 4 | 2.8 | 12.9 | 173 | 118.8 | 52.5 | 69.4 | 71.8 | 39.4 | Woodland | Carnivore | Invertivore | Aerial | 0 | 2 | 3 | close | rock-face |
| Stachyris_striolata | 18 | 10.8 | 3.8 | 6 | 22.9 | 66.4 | 5.2 | 61.6 | 7.7 | 55.2 | 28 | Forest | Carnivore | Invertivore | Insessorial | 0 | 4 | 4 | open | bush |
| Glaucidium_cuculoides | 20.7 | 13.4 | 8 | 13.4 | 23.4 | 147.8 | 34.5 | 113.2 | 23.3 | 81 | 198.2 | Forest | Carnivore | Invertivore | Insessorial | 1 | 4 | 4 | close | canopy |
| Lonchura_punctulata | 11.8 | 8.8 | 6.7 | 7.5 | 14 | 52.6 | 11.9 | 42.4 | 22 | 39 | 13.6 | Grassland | Herbivore | Granivore | Generalist | 0 | 4 | 8 | open | bush |
| Rostratula_benghalensis | 52 | 39.8 | 4.3 | 6.7 | 44.4 | 127.6 | 38.5 | 83.5 | 31.6 | 42 | 121 | Wetland | Omnivore | Aquatic predator | Terrestrial | 0 | 4 | 5 | open | groud |
| Parus_major | 11.3 | 8 | 4 | 4.3 | 17.4 | 70.9 | 13.3 | 57.7 | 18.7 | 61.2 | 16.3 | Woodland | Omnivore | Invertivore | Insessorial | 0 | 6 | 9 | close | hole |
| Aethopyga_christinae | 17.4 | 12.4 | 3.5 | 3.5 | 13.9 | 47.8 | 8.8 | 39 | 18.2 | 46 | 5.2 | Forest | Herbivore | Omnivore | Insessorial | 0 | 2 | 3 | open | canopy |
| Ardeola_bacchus | 72.9 | 52.4 | 9.2 | 12.2 | 56.2 | 213 | 56.6 | 153.4 | 26.8 | 73.4 | 304.9 | Wetland | Carnivore | Aquatic predator | Terrestrial | 0 | 3 | 3 | open | canopy |
| Pericrocotus_flammeus | 18.7 | 10.7 | 6.1 | 5.5 | 17 | 92.1 | 25.9 | 66.1 | 28.1 | 88.4 | 23.3 | Forest | Carnivore | Invertivore | Insessorial | 0 | 2 | 4 | open | canopy |
| Prinia_inornata | 12.2 | 6.7 | 2.3 | 2.6 | 18.1 | 50.4 | 7.2 | 43.4 | 14.2 | 70.8 | 7 | Shrubland | Carnivore | Invertivore | Generalist | 0 | 4 | 6 | open | reed |
| Dicaeum_concolor | 10.9 | 7.1 | 2.7 | 3 | 11.8 | 47.2 | 9.5 | 37.6 | 20.3 | 26.2 | 6.2 | Forest | Herbivore | Omnivore | Insessorial | 0 | 2 | 3 | close | canopy |
| Dendrocopos_major | 27.9 | 21.8 | 9.3 | 8.7 | 23.9 | 136.2 | 40.8 | 98.5 | 29.3 | 88 | 74.9 | Woodland | Omnivore | Omnivore | Insessorial | 0 | 4 | 6 | close | canopy |
| Upupa_epops | 55.8 | 46.9 | 5.5 | 6.3 | 21.1 | 142.3 | 30.5 | 111.5 | 21.4 | 96.9 | 66.9 | Grassland | Carnivore | Invertivore | Terrestrial | 0 | 6 | 8 | close | canopy |
| Alcippe_morrisonia | 12.3 | 6.7 | 3 | 3.8 | 19.2 | 60.9 | 8.2 | 53.5 | 13.2 | 52.4 | 15.3 | Forest | Omnivore | Invertivore | Insessorial | 0 | 3 | 4 | open | bush |
| Acrocephalus_arundinaceus | 23 | 12.8 | 4.7 | 5.4 | 28.1 | 95.4 | 27.2 | 68.1 | 28.6 | 80.8 | 30 | Wetland | Carnivore | Invertivore | Insessorial | 0 | 3 | 6 | open | reed |
| Delichon_nipalense | 8.7 | 4.2 | 3.2 | 3 | 10.8 | 92 | 49.8 | 42.2 | 54.1 | 43.2 | 15 | Rock | Carnivore | Invertivore | Aerial | 0 | 4 | 4 | close | rock-face |
| Gallicrex_cinerea | 42.8 | 17.9 | 5.8 | 9.5 | 64.4 | 185.9 | 58.1 | 125.7 | 31.6 | 73.2 | 390.4 | Wetland | Herbivore | Omnivore | Terrestrial | 0 | 3 | 8 | open | bush |
| Culicicapa_ceylonensis | 11.7 | 5.9 | 3.8 | 2.5 | 12.2 | 58.4 | 11 | 47.2 | 18.9 | 48.4 | 7.7 | Woodland | Carnivore | Invertivore | Insessorial | 0 | 3 | 3 | open | rock-face |
| Pernis_ptilorhyncus | 55.9 | 21.4 | 13.1 | 15.4 | 49 | 413.6 | 130.7 | 278.6 | 32 | 263.8 | 1141.1 | Woodland | Carnivore | Invertivore | Insessorial | 0 | 2 | 2 | open | canopy |
| Accipiter_trivirgatus | 31.7 | 17.3 | 10.5 | 14.1 | 56.9 | 216.2 | 49.6 | 166.1 | 23 | 171.9 | 265 | Forest | Carnivore | Vertivore | Insessorial | 0 | 2 | 3 | open | canopy |
| Dicrurus_aeneus | 21.6 | 13.3 | 7.6 | 6.1 | 16 | 116.9 | 34.4 | 82.5 | 29.4 | 84.6 | 26 | Forest | Carnivore | Invertivore | Insessorial | 0 | 3 | 4 | open | canopy |
| Cyornis_hainanus | 13.9 | 8.7 | 4.3 | 3.5 | 16.2 | 68.4 | 14.7 | 52.7 | 21.7 | 59 | 14.4 | Forest | Carnivore | Invertivore | Insessorial | 0 | 3 | 3 | close | groud |
| Arborophila_ardens | 21.9 | 9.9 | 5.3 | 7.9 | 38.4 | 130.3 | 34.1 | 96.1 | 26.2 | 42.1 | 266.6 | Forest | Omnivore | Omnivore | Terrestrial | 0 | 2 | 2 | open | groud |
| Centropus_sinensis | 46.6 | 29.3 | 9.9 | 16.3 | 55.6 | 212.4 | 30.9 | 181.9 | 14.6 | 260.9 | 280.7 | Woodland | Carnivore | Vertivore | Terrestrial | 0 | 3 | 5 | open | bush |
| Alcippe_brunnea | 13.8 | 7.3 | 3 | 4.1 | 22.6 | 59.1 | 6 | 52.9 | 10.1 | 48.8 | 17.4 | Forest | Carnivore | Invertivore | Generalist | 0 | 2 | 3 | open | bush |
| Accipiter_badius | 20.6 | 12.1 | 8.8 | 11.6 | 43 | 186.7 | 62.5 | 127.4 | 32.9 | 140.6 | 131.2 | Shrubland | Carnivore | Vertivore | Insessorial | 0 | 3 | 4 | open | canopy |
| Phylloscopus_fuscatus | 11.9 | 7 | 2.5 | 2.6 | 21.4 | 62.3 | 12.1 | 51.1 | 19.1 | 53.4 | 8.7 | Wetland | Carnivore | Invertivore | Insessorial | 0 | 5 | 5 | open | bush |
| Elanus_caeruleus | 30.9 | 16 | 9.2 | 11.3 | 32.1 | 286.3 | 132.1 | 160.2 | 45.2 | 127.7 | 259.8 | Grassland | Carnivore | Vertivore | Aerial | 0 | 3 | 5 | open | canopy |
| Hypsipetes_leucocephalus | 25.2 | 14.3 | 4.9 | 5.7 | 18 | 117.8 | 29.1 | 88.7 | 24.7 | 97.8 | 51.8 | Forest | Herbivore | Frugivore | Insessorial | 0 | 2 | 4 | open | canopy |
| Garrulax_chinensis | 26 | 16 | 5.6 | 8 | 39.2 | 113 | 6.2 | 106.8 | 5.5 | 117.5 | 88.5 | Forest | Carnivore | Invertivore | Generalist | 0 | 4 | 4 | open | bush |
| Dicrurus_macrocercus | 25.4 | 15.4 | 8.4 | 8.4 | 22.2 | 141 | 39.2 | 101.2 | 27.8 | 145.6 | 48.3 | Shrubland | Carnivore | Invertivore | Insessorial | 0 | 3 | 4 | open | canopy |
| Sturnus_nigricollis | 32.4 | 20.1 | 6 | 7.9 | 41.7 | 158.1 | 41.3 | 116 | 26.2 | 95.4 | 156.9 | Grassland | Carnivore | Invertivore | Insessorial | 0 | 4 | 6 | open | canopy |
| Garrulax_pectoralis | 33.1 | 22.1 | 7.4 | 9.3 | 46.2 | 141.6 | 9.8 | 130.2 | 7 | 135.5 | 145.1 | Forest | Carnivore | Invertivore | Terrestrial | 0 | 4 | 4 | open | bush |
| Megalaima_faber | 24.3 | 17.4 | 8.8 | 11.2 | 25 | 103.2 | 20.8 | 82.5 | 20.1 | 63.5 | 87.7 | Forest | Herbivore | Frugivore | Insessorial | 0 | 3 | 3 | close | canopy |
| Milvus_migrans | 40 | 22.4 | 13 | 17.4 | 51.4 | 457.4 | 219.9 | 237.5 | 48.1 | 269.5 | 734.1 | Woodland | Omnivore | Omnivore | Aerial | 0 | 2 | 3 | open | canopy |
| Hypothymis_azurea | 11.8 | 8.5 | 4.6 | 3.7 | 15.8 | 69.8 | 11.1 | 58.6 | 16 | 75.2 | 11.1 | Forest | Carnivore | Invertivore | Insessorial | 0 | 3 | 5 | open | bush |
| Stachyris_ruficeps | 15 | 8.9 | 2.6 | 3.3 | 19.6 | 55 | 5.4 | 50 | 9.8 | 47 | 10.3 | Shrubland | Carnivore | Invertivore | Insessorial | 0 | 4 | 5 | open | bush |
| Lanius_cristatus | 19.3 | 11.6 | 6.2 | 7.9 | 23.8 | 85 | 22.5 | 62.2 | 26.6 | 79.9 | 32.2 | Shrubland | Carnivore | Invertivore | Insessorial | 0 | 5 | 7 | open | canopy |
| Porzana_fusca | 26 | 12.1 | 3 | 5.1 | 31 | 94.5 | 21 | 73.5 | 22.2 | 42.5 | 57.3 | Wetland | Omnivore | Aquatic predator | Terrestrial | 0 | 5 | 9 | open | groud |
| Gallus_gallus | 30.9 | 15.4 | 8.9 | 8.9 | 68.1 | 218.2 | 48.9 | 167.6 | 22.5 | 194 | 751.7 | Forest | Herbivore | Omnivore | Terrestrial | 0 | 6 | 8 | open | groud |
| Urocissa_erythrorhyncha | 38.7 | 22.5 | 12.1 | 13 | 45.3 | 181.3 | 35 | 146.6 | 19.3 | 366.3 | 151.5 | Forest | Carnivore | Omnivore | Insessorial | 0 | 4 | 5 | open | canopy |
| Treron_curvirostra | 19.4 | 11.9 | 5.9 | 7.4 | 19.9 | 135.9 | 47.5 | 88.6 | 34.9 | 73.9 | 134.5 | Forest | Herbivore | Frugivore | Insessorial | 0 | 2 | 2 | open | bush |
| Garrulax_canorus | 23.9 | 13.6 | 5.2 | 7 | 36.4 | 92.4 | 6.4 | 86 | 7 | 104.1 | 62.8 | Shrubland | Carnivore | Invertivore | Terrestrial | 0 | 4 | 4 | open | bush |
| Nectarinia_jugularis | 20.7 | 14.9 | 3.3 | 2.5 | 14.5 | 53.4 | 8.8 | 40.5 | 17.7 | 35.2 | 9 | Shrubland | Omnivore | Omnivore | Insessorial | 0 | 3 | 3 | open | canopy |
| Prinia_flaviventris | 14 | 9.5 | 2.9 | 2.6 | 20.9 | 45.4 | 5.3 | 39.6 | 11.9 | 62.6 | 7 | Shrubland | Carnivore | Invertivore | Insessorial | 0 | 4 | 5 | open | reed |
| Phylloscopus_inornatus | 9.9 | 5.3 | 2.5 | 2.3 | 16.8 | 58.4 | 11.3 | 48.2 | 19 | 37.8 | 6.6 | Woodland | Carnivore | Invertivore | Insessorial | 0 | 5 | 6 | open | groud |
| Ixobrychus_sinensis | 62.4 | 43.7 | 6 | 8.2 | 42.4 | 129.4 | 39.6 | 88.4 | 31 | 40.8 | 94.3 | Wetland | Carnivore | Aquatic predator | Terrestrial | 0 | 7 | 7 | open | bush |
| Otus_spilocephalus | 17.9 | 9.6 | 5.1 | 9.2 | 28.4 | 140.4 | 35.8 | 105 | 25.4 | 76.8 | 67.5 | Forest | Carnivore | Invertivore | Insessorial | 1 | 3 | 4 | close | canopy |
| Sturnus_sinensis | 20.4 | 12.9 | 5.1 | 5.1 | 25.6 | 101.8 | 32.6 | 68.4 | 32.3 | 57.5 | 61 | Grassland | Carnivore | Invertivore | Generalist | 0 | 4 | 5 | close | canopy |
| Motacilla_cinerea | 17.4 | 10.8 | 3.1 | 3 | 18.2 | 82.8 | 28.6 | 54 | 34.6 | 94.4 | 17.2 | Riverine | Carnivore | Invertivore | Terrestrial | 0 | 5 | 5 | open | groud |
| Artamus_fuscus | 20.9 | 13.4 | 7.4 | 7.9 | 17.4 | 131.9 | 63.9 | 68.2 | 48.4 | 54.9 | 39.8 | Woodland | Carnivore | Invertivore | Aerial | 0 | 2 | 2 | close | bush |
| Actitis_hypoleucos | 30.1 | 19.9 | 3.1 | 4 | 22.4 | 109.9 | 51.1 | 55.4 | 48 | 57.4 | 48 | Wetland | Carnivore | Aquatic predator | Terrestrial | 0 | 4 | 5 | open | groud |
| Acridotheres_tristis | 26.8 | 16 | 6.4 | 8 | 38.6 | 139.8 | 32.8 | 106.3 | 23.6 | 84.8 | 116.4 | Human Modified | Carnivore | Omnivore | Terrestrial | 0 | 4 | 6 | close | canopy |
| Hirundo_rustica | 12.4 | 6 | 4.5 | 2.7 | 11.7 | 115.8 | 62.3 | 56 | 52.6 | 71.4 | 17.9 | Human Modified | Carnivore | Invertivore | Aerial | 0 | 4 | 5 | close | rock-face |
| Charadrius_dubius | 16.1 | 9.3 | 2.6 | 3.1 | 23.2 | 114.8 | 57.2 | 55 | 51 | 55.6 | 38.7 | Wetland | Carnivore | Aquatic predator | Terrestrial | 0 | 3 | 4 | open | groud |
| Anthus_richardi | 18 | 11.3 | 4 | 4.3 | 29.2 | 92 | 26.6 | 64.3 | 29.3 | 71.6 | 32.2 | Grassland | Carnivore | Invertivore | Terrestrial | 0 | 5 | 5 | open | groud |
| Hemixos_castanonotus | 19.8 | 11.4 | 4.6 | 5 | 17.4 | 97.4 | 19.2 | 78.2 | 19.7 | 89.9 | 35.3 | Forest | Omnivore | Frugivore | Insessorial | 0 | 3 | 5 | open | canopy |
| Garrulax_castanotis | 26.5 | 15.8 | 5 | 7.3 | 40.9 | 115.2 | 6.4 | 106.6 | 5.6 | 116 | 97.5 | Forest | Carnivore | Invertivore | Terrestrial | 0 | 2 | 3 | open | bush |
| Seicercus_castaniceps | 9.6 | 4.9 | 2.5 | 2.4 | 15.4 | 48.8 | 9.5 | 39.3 | 19.3 | 37.2 | 5.3 | Forest | Carnivore | Invertivore | Insessorial | 0 | 4 | 5 | open | groud |
| Ixobrychus_cinnamomeus | 58.7 | 40.6 | 6.6 | 10.6 | 46.9 | 144.3 | 36.1 | 105.1 | 25.6 | 40.4 | 126.5 | Human Modified | Carnivore | Aquatic predator | Terrestrial | 0 | 3 | 6 | open | groud |
| Otus_lettia | 21.2 | 12 | 6.6 | 10.6 | 33.8 | 155.8 | 39.8 | 116 | 25.6 | 81.2 | 139 | Forest | Carnivore | Invertivore | Insessorial | 1 | 3 | 4 | close | canopy |
| Glaucidium_brodiei | 20.5 | 11.3 | 6.7 | 10.6 | 16.7 | 92.6 | 19.4 | 74.3 | 20.7 | 50.4 | 59.4 | Forest | Carnivore | Vertivore | Insessorial | 1 | 4 | 4 | close | canopy |
| Chalcophaps_indica | 20.9 | 11.3 | 3.1 | 4.2 | 25.8 | 143.9 | 40 | 100.1 | 28.7 | 83.9 | 136.5 | Forest | Herbivore | Omnivore | Generalist | 0 | 2 | 2 | open | canopy |
| Phaenicophaeus_tristis | 35.6 | 22.3 | 9.4 | 12.6 | 32.8 | 165.7 | 27 | 141 | 16 | 333.3 | 117 | Forest | Carnivore | Invertivore | Insessorial | 0 | 2 | 4 | open | bush |
| Passer_montanus | 13.2 | 8.2 | 5.5 | 6.5 | 16.4 | 67.9 | 16.7 | 51.2 | 24.6 | 51 | 21.4 | Woodland | Omnivore | Granivore | Terrestrial | 0 | 5 | 6 | close | hole |
| Phylloscopus_coronatus | 13 | 7.4 | 3.2 | 3.1 | 16.6 | 61.3 | 13.8 | 47.5 | 22.6 | 47.4 | 10.1 | Woodland | Carnivore | Invertivore | Insessorial | 0 | 4 | 7 | open | groud |
| Bubulcus_ibis | 63.3 | 43.5 | 8.6 | 11.5 | 76.8 | 244.4 | 73 | 171.2 | 29.9 | 90.9 | 366 | Human Modified | Carnivore | Omnivore | Terrestrial | 0 | 5 | 7 | open | canopy |
| Alcedo_atthis | 42.2 | 32.5 | 5.7 | 7.4 | 9.9 | 73.3 | 19.3 | 52.6 | 26.9 | 32.3 | 31.1 | Riverine | Carnivore | Aquatic predator | Insessorial | 0 | 5 | 7 | close | rock-face |
| Phalacrocorax_carbo | 82.7 | 57 | 13.2 | 18.3 | 64.2 | 340.2 | 124.2 | 211.7 | 37.1 | 148.2 | 2529 | Wetland | Carnivore | Aquatic predator | Aquatic | 0 | 3 | 5 | open | canopy |
| Copsychus_saularis | 23.2 | 15 | 5.4 | 5.8 | 29.5 | 97.5 | 18.7 | 79.2 | 19.1 | 84 | 36 | Woodland | Carnivore | Invertivore | Terrestrial | 0 | 5 | 5 | close | canopy |
| Streptopelia_orientalis | 21.9 | 8.3 | 3.1 | 4 | 25.5 | 176.5 | 57.2 | 117.7 | 32.8 | 127.2 | 232.9 | Woodland | Herbivore | Omnivore | Terrestrial | 0 | 2 | 2 | open | canopy |
| Spilornis_cheela | 45.6 | 30.1 | 14.4 | 20.8 | 82.2 | 373.7 | 112.6 | 261.1 | 30.1 | 237.4 | 597.7 | Forest | Carnivore | Vertivore | Insessorial | 0 | 1 | 1 | open | canopy |
| Anthus_hodgsoni | 14.1 | 7.9 | 3.4 | 3.4 | 20.2 | 81 | 20.8 | 59.5 | 25.9 | 58.4 | 21.3 | Grassland | Carnivore | Invertivore | Terrestrial | 0 | 5 | 5 | open | groud |
| Sturnus_sericeus | 26.8 | 16.3 | 4.8 | 5.5 | 29.9 | 120.6 | 39.2 | 80.8 | 32.6 | 71.8 | 65 | Shrubland | Omnivore | Invertivore | Insessorial | 0 | 6.3 | 6.3 | close | canopy |
| Cuculus_micropterus | 30.3 | 19.4 | 8.4 | 9.3 | 19.5 | 196.3 | 87.9 | 107 | 45.1 | 146.1 | 88.8 | Forest | Carnivore | Invertivore | Insessorial | 0 | 1 | 1 | parasitism | parasitism |
| Apus_nipalensis | 8.9 | 3.5 | 3.2 | 2.1 | 11.3 | 130 | 89 | 42.7 | 67.6 | 48 | 29.8 | Human Modified | Carnivore | Invertivore | Aerial | 0 | 2 | 4 | close | rock-face |
| Emberiza_pusilla | 10.7 | 6.8 | 3.7 | 4.6 | 18 | 70.3 | 17.1 | 52.9 | 24.5 | 55.5 | 14.6 | Forest | Herbivore | Granivore | Generalist | 0 | 4 | 6 | open | groud |
| Centropus_bengalensis | 28.2 | 15.7 | 6.6 | 11 | 39.3 | 154.6 | 29.6 | 129.6 | 18.6 | 185.2 | 148.9 | Grassland | Carnivore | Invertivore | Terrestrial | 0 | 3 | 5 | open | bush |
| Alauda_gulgula | 14.8 | 9.7 | 4.3 | 4.6 | 23.3 | 86.4 | 23.7 | 62.6 | 27.5 | 52.2 | 26.3 | Grassland | Omnivore | Omnivore | Terrestrial | 0 | 3 | 5 | open | groud |
| Dendrocopos_canicapillus | 16.8 | 13.7 | 5.1 | 5.1 | 14.2 | 83.3 | 23.6 | 60 | 28.3 | 42.3 | 25.8 | Forest | Carnivore | Invertivore | Insessorial | 0 | 4 | 5 | close | canopy |
| Nycticorax_nycticorax | 80.5 | 56.7 | 12.1 | 18.6 | 69.2 | 285.7 | 92.7 | 187.8 | 33.1 | 95.8 | 810 | Wetland | Carnivore | Aquatic predator | Terrestrial | 0 | 4 | 4 | open | canopy |
| Cuculus_sparverioides | 30.3 | 17.2 | 7.8 | 8.2 | 24.6 | 235 | 77 | 158.5 | 32.7 | 207.6 | 151 | Woodland | Carnivore | Invertivore | Insessorial | 0 | 1 | 2 | parasitism | parasitism |
| Eudynamys_scolopaceus | 32.1 | 20.4 | 10.3 | 10.6 | 31 | 196.4 | 62.4 | 136.4 | 31.4 | 179.7 | 194.9 | Woodland | Herbivore | Frugivore | Insessorial | 0 | 1 | 1 | parasitism | parasitism |
| Caprimulgus_macrurus | 22.3 | 7.7 | 5.1 | 3.2 | 13 | 179.2 | 84.5 | 94.7 | 47.1 | 124.8 | 66.2 | Woodland | Carnivore | Invertivore | Generalist | 1 | 2 | 2 | open | groud |
| Pomatorhinus_hypoleucos | 41.3 | 30.3 | 5.4 | 7.3 | 37.3 | 105.4 | 6.5 | 97.7 | 6.2 | 93.8 | 75.6 | Shrubland | Carnivore | Invertivore | Terrestrial | 0 | 2 | 3 | open | bush |
| Francolinus_pintadeanus | 26.9 | 14.7 | 8.8 | 10.2 | 37.9 | 149.4 | 37.4 | 112.6 | 25 | 72.4 | 310 | Forest | Herbivore | Omnivore | Terrestrial | 0 | 3 | 6 | open | groud |
| Dicaeum_cruentatum | 11 | 7.3 | 3.2 | 3 | 12 | 47.3 | 10.2 | 37.6 | 21.3 | 25 | 5.6 | Forest | Herbivore | Omnivore | Insessorial | 0 | 2 | 3 | close | canopy |
| Stigmatopelia_chinensis | 19.9 | 10.8 | 3.5 | 3.5 | 19.9 | 138.8 | 42 | 96.7 | 30.3 | 127.3 | 159 | Woodland | Herbivore | Granivore | Terrestrial | 0 | 2 | 2 | open | bush |
| Lanius_schach | 21.8 | 13.2 | 6.8 | 9.2 | 29.2 | 92.4 | 18.6 | 73.2 | 20.3 | 123.3 | 51.5 | Shrubland | Carnivore | Invertivore | Insessorial | 0 | 4 | 5 | open | canopy |
| Pomatorhinus_ruficollis | 22.5 | 15.6 | 3.3 | 5.4 | 28 | 78.8 | 7.7 | 69.8 | 10 | 85.4 | 31.6 | Forest | Carnivore | Invertivore | Generalist | 0 | 4 | 4 | open | bush |
| Cisticola_juncidis | 12.1 | 6.8 | 2.3 | 2.6 | 17.7 | 48.9 | 6.7 | 42.8 | 13.5 | 40.3 | 6.8 | Grassland | Carnivore | Invertivore | Generalist | 0 | 4 | 5 | open | reed |

**Table S4**. Models selection of multiple linear regression between ses.MFPD and environmental data, interspecific competition. “Pr (>|t|)” in bold means the variables is significant.

| Model | Variable | Estimate | Std. Error | t value | Pr (>\|t\|) |
| --- | --- | --- | --- | --- | --- |
| Fit1 | (Intercept) | 844.742689 | 405.830506 | 2.08151599 | 0.28511687 |
|  | ALT | -0.0128284 | 0.0088276 | -1.4532172 | 0.38369959 |
|  | TEMP | -0.289378 | 0.13508142 | -2.1422489 | 0.27803474 |
|  | PREC | -0.0003119 | 4.8149E-05 | -6.4785791 | 0.0974959 |
|  | NDVI | 0.05039319 | 0.00985669 | 5.11258622 | 0.12296764 |
|  | NPP | -0.0105113 | 0.00257704 | -4.0788429 | 0.15305953 |
|  | HII | 0.3284005 | 0.01958773 | 16.7656232 | **0.0379268** |
|  | COMP | 9.80091833 | 4.29232538 | 2.28335866 | 0.26278978 |
| Fit2 | (Intercept) | -24.6321005 | 4.008995111 | -6.144208166 | **0.025481056** |
|  | ALT | 0.005987881 | 0.001474327 | 4.061434577 | 0.055614416 |
|  | PREC | -0.000232724 | 5.15547E-05 | -4.514129395 | **0.045733806** |
|  | NDVI | 0.058132546 | 0.015330835 | 3.791870836 | 0.063043711 |
|  | NPP | -0.009367 | 0.004214494 | -2.222568178 | 0.156313066 |
|  | HII | 0.293377935 | 0.018036318 | 16.26595462 | **0.003758263** |
|  | COMP | 17.31531951 | 4.13557763 | 4.186916812 | 0.052585163 |
| Fit3 | (Intercept) | -24.22031424 | 6.090940914 | -3.976448726 | **0.028444388** |
|  | ALT | 0.005498042 | 0.002217172 | 2.479754644 | 0.089289351 |
|  | PREC | -0.000250363 | 7.74772E-05 | -3.23143595 | **0.048163227** |
|  | NDVI | 0.059773019 | 0.023290298 | 2.566434311 | 0.082749331 |
|  | HII | 0.304257639 | 0.026402581 | 11.52378403 | **0.001402929** |
|  | COMP | 14.0454903 | 5.8785193 | 2.389290497 | 0.096803246 |
| Fit4 | (Intercept) | -10.697267 | 4.730010337 | -2.261573705 | 0.086529732 |
|  | ALT | 0.009941906 | 0.002143809 | 4.637495377 | **0.009752278** |
|  | PREC | -7.49707E-05 | 5.65064E-05 | -1.326765399 | 0.255260091 |
|  | HII | 0.276200614 | 0.037205613 | 7.423627494 | **0.001757498** |
|  | COMP | 7.627840776 | 8.236172508 | 0.926139025 | 0.406793009 |
| Fit5 | (Intercept) | -11.86475109 | 4.988287729 | -2.378521797 | 0.063280733 |
|  | ALT | 0.01004085 | 0.002299646 | 4.366258448 | **0.007247493** |
|  | HII | 0.276076894 | 0.039934191 | 6.913296213 | **0.000970811** |
|  | COMP | 7.012148604 | 8.826179892 | 0.794471526 | 0.462945379 |
| Fit6 | (Intercept) | -7.988941947 | 1.008413781 | -7.922285572 | **0.000214781** |
|  | ALT | 0.010794341 | 0.002029556 | 5.318573441 | **0.001797627** |
|  | HII | 0.2819917 | 0.038009105 | 7.419056643 | **0.000308394** |
